# Endogenous oxytocin, cortisol, and testosterone in response to group singing

**DOI:** 10.1016/j.yhbeh.2021.105105

**Published:** 2022-01-06

**Authors:** D.L. Bowling, J. Gahr, P. Graf Ancochea, M. Hoeschele, V. Canoine, L. Fusani, W.T. Fitch

**Affiliations:** a Department of Psychiatry and Behavioral Sciences, Stanford University School of Medicine, United States of America; b Department of Behavioral & Cognitive Biology, University of Vienna, Austria; c Acoustics Research Institute, Austrian Academy of Sciences, Austria; d Konrad Lorenz Institute of Ethology, University of Veterinary Medicine, Vienna, Austria; e CogSci Hub University of Vienna, Austria

**Keywords:** Speech, Music, Synchrony, Bonding, Affect, Oxytocin, Cortisol, Testosterone

## Abstract

Humans have sung together for thousands of years. Today, regular participation in group singing is associated with benefits across psychological and biological dimensions of human health. Here we examine the hypothesis that a portion of these benefits stem from changes in endocrine activity associated with affiliation and social bonding. Working with a young adult choir (*n* = 71), we measured changes salivary concentrations of oxytocin, cortisol, and testosterone from before and after four experimental conditions crossing two factors: vocal production mode (singing vs. speaking) and social context (together vs. alone). Salivary oxytocin and cortisol decreased from before to after the experimental manipulations. For oxytocin the magnitude of this decrease was significantly smaller after singing compared to speaking, resulting in concentrations that were significantly elevated after singing together compared to speaking together, after controlling for baseline differences. In contrast, the magnitude of the salivary cortisol decreases was the same across experimental manipulations, and although large, could not be separated from diurnal cycling. No significant effects were found in a low-powered exploratory evaluation of testosterone (tested only in males). At a psychological level, we found that singing stimulates greater positive shifts in self-perceived affect compared to speaking—particularly when performed together—and that singing together enhances feelings of social connection more than speaking together. Finally, measurements of heart rate made for a subset of participants provide preliminary evidence regarding physical exertion levels across conditions. These results are discussed in the context of a growing multidisciplinary literature on the endocrinological correlates of musical behavior. We conclude that singing together can have biological and psychological effects associated with affiliation and social bonding, and that these effects extend beyond comparable but non-musical group activities. However, we also note that these effects appear heavily influenced by broader contextual factors that shape social dynamics, such as stress levels, the intimacy of interactions, and the status of existing relationships.

## 1. Introduction

Singing together with others (“group singing”) is perhaps the oldest and most widely conserved form of musical behavior (Jordania, 2015). Over the last several decades, researchers have sought to understand its ubiquity by studying its effects on health and wellbeing. The origins of this work focused primarily on singing's capacity to improve quality of life, particularly for vulnerable populations like inmates or the unhoused (e.g., Bailey and Davidson, 2002; Silber, 2005). Early studies explored the efficacy of singing-based interventions, using semi-structured interviews and questionnaires to assess psychological responses to regular participation in choir practice. These studies associate a wide array of benefits with group singing (Clift et al., 2008). For example, in one influential study focused on adults aged 65+, an eight-month intervention of weekly choir practice compared against a no-treatment control showed significant improvements in mood, decreases in loneliness, and reductions in number of doctor visits for the intervention group (Cohen et al., 2006). Similar benefits have been reported by users of mental health services (Clift and Morrison, 2011; Dingle et al., 2013), as well as members of the broader general public (Clift et al., 2010). Psychologically, the benefits of group singing appear to be associated with group singing's capacity to induce positive affect, reduce stress, and, of particular interest here, improve social functioning (Clift et al., 2008).

Despite the clear picture of benefits associated with group singing, a number of important questions remain. One concerns the degree to which such benefits are specific to singing, rather than group activity or recreation more generally. Most early exploratory studies did not include the experimental control conditions required to address this question. When control conditions have been included, they are often characterized by marked differences in physical activity that confound the role of group singing in driving observed effects (cf. Tarr et al., 2015). Examples include comparisons of group singing with group music listening (Kreutz et al., 2004; Unwin et al., 2002), quiet chatting (Kreutz, 2014), and swimming (Valentine and Evans, 2001). In an early acknowledgement of this confound, Anshel and Kipper (1988) independently manipulated music and physical activity across four conditions: group singing (music/activity), group music listening (music/no activity), group poetry reading (no music/activity), and group film watching (no music/no activity). Their results showed independent effects of each factor, with music increasing feelings of trust, and physical activity increasing cooperative behavior in a Prisoner's Dilemma game. This was one of the first experimental indications that musical behaviors—particularly active interpersonal kinds like group singing—can be potent promoters of prosociality (Gaston, 1968; Roederer, 1984; Savage et al., 2021).

Academic appreciation for music's capacity to stimulate affiliation and social bonding has only grown since 1988, with many recent studies focusing on it as a potential key element of music's evolutionary origins (e.g., Bowling et al., 2013; Freeman, 2001; Hove and Risen, 2009; Kirschner and Tomasello, 2010; Kokal et al., 2011; Kreutz, 2014; Launay et al., 2016; Pearce et al., 2015; Ravignani et al., 2014; Reddish et al., 2013a, 2013b; Tarr et al., 2014, 2015; Weinstein et al., 2016; Welch et al., 2018). A recent synthesis has brought together ideas on music's role in stimulating social cohesion under a unified “music and social bonding” (MSB) hypothesis (Savage et al., 2021). The MSB hypothesis proposes that the capacity of music to promote the formation, strengthening, and maintenance of affiliative connections (or “social bonds”) provides the ultimate adaptive basis for the evolution of music (but see Mehr et al., 2021; Bowling et al., 2021). In keeping with the MSB hypothesis, interpersonal synchrony—i.e., temporally-coordinated activity between individuals, as occurs in group singing—increases affiliative feelings and behaviors more than asynchronous or uncoodinated behaviors (reviewed in Mogan et al., 2017; Rennung and Göritz, 2016). This converges with ethnographic and historical evidence associating music with social contexts that build cultural and community identity, such as celebrations and festivals, religious worship, military training, and political activity (McNeill, 1995; Mehr et al., 2018, 2019; Savage et al., 2015). The capacity of group singing to stimulate affiliation and social bonding thus has implications that extend beyond health and wellbeing, potentially to the biological foundations of music.

Moving towards these foundations, a second set of unanswered questions is focused on the biological mechanisms by which the benefits of group singing are realized. Studies addressing these mechanisms have largely focused on biochemical markers of immune and endocrine function, mostly measured in saliva sampled in temporal proximity to group singing. For example, at least three studies have reported effects of group singing on immune function, including increased salivary antibody content and upregulated cytokine networks (Beck et al., 2000; Fancourt et al., 2016; Kreutz et al., 2004). More relevant here, a handful of studies have focused on social aspects of endocrine function by measuring the hypophyseal neuropeptide oxytocin, as we do here. Oxytocin is an essential modulator of social behavior and cognition in eutherian mammals (Jurek and Neumann, 2018). In addition to its roles in core reproductive functions like sex, parturition, and lactation (Blaicher et al., 1999; Carmichael et al., 1994; Fuchs and Fuchs, 1984; Insel and Shapiro, 1992; Uvnäs-Moberg and Prime, 2013), preclinical studies manipulating oxytocin in the brains of non-human animals indicate more subtle effects on social behavior and perception, e.g., in the context of affiliative behavior, partner preferences, pair-bonding, social touch, and stress reactivity (Dölen et al., 2013; Donaldson and Young, 2008; Hung et al., 2017; Menon et al., 2018; Neumann et al., 2000; Ross et al., 2009; Tang et al., 2020). Studies of oxytocin in humans provide evidence of similar functions. For example, short bouts of focused interpersonal synchrony stimulate endogenous increases in salivary oxytocin levels (Spengler et al., 2017). Likewise, manipulation of oxytocin with intranasal administration suggests that oxytocin can act to increase sensitivity to the emotional status of others (Shahrestani et al., 2013), trust (Kosfeld et al., 2005; but see Declerck et al., 2020), and cooperation (De Dreu et al., 2010; Declerck et al., 2010). Although oxytocin is widely considered to be a promoter of prosociality, its relationship with social cognition and behavior is highly sensitive to context (Bartz et al., 2011). Taking trust as an example, the directionality of oxytocin's effect depend on who a person is interacting with (friend, foe, or stranger; De Dreu et al., 2010; Declerck et al., 2010), as well as individual differences in stress and anxiety associated with social attachment (Bartz et al., 2010; Declerck et al., 2020). The context-sensitivity of the oxytocin system is underscored by existing studies of oxytocin and group singing, with two reporting increases in oxytocin concentrations after group singing (Keeler et al., 2015; Kreutz, 2014), and two reporting decreases (Fancourt et al., 2016; Schladt et al., 2017; see Discussion for details). Importantly here, Schladt et al. (2017) made a controlled comparison of singing together versus singing alone (as we do), finding that only singing together had a significant effect on salivary oxytocin concentrations. The directionality of oxytocin changes associated with group singing thus remains equivocal, with some evidence supporting its sensitivity to social context.

In addition to oxytocin, several biologically-oriented studies of group singing have examined the glucocorticoid hormone cortisol, as we also do here. As a major end-product of hypothalamo-pituitary-adrenocortical (HPA) axis activation, cortisol levels provide insight into acute physical and/or psychosocial stress (Edwards et al., 2001; Schmidt-Reinwald et al., 1999), and inversely, an individual’s capacity for affiliation and social bonding (Beery and Kaufer, 2015; Gaab et al., 2005). Existing studies primarily show decreases in salivary cortisol concentrations after group singing, particularly in relatively low-stress settings such as a choir practice (Beck et al., 2000; Fancourt et al., 2015, 2016; Schladt et al., 2017). Like oxytocin, cortisol responses to group singing are highly sensitive to context. Group singing in relatively high-stress settings, like live performance, has been associated with cortisol increases (Beck et al., 2000; Fancourt et al., 2015). Regarding social context, Schladt et al. (2017) found that salivary cortisol concentrations decrease more after singing together than they do after singing alone.

Finally, in the current study we also conducted an exploratory analysis of the primary sex hormone testosterone, which has not previously been studied in the context of group singing. Traditionally implicated in social forms of aggression (e.g., dominance; Rose et al., 1971), recent studies suggest that the behavioral effects of testosterone may be best understood in terms of supporting a drive to seek and maintain social status (Eisenegger et al., 2011). Acute testosterone spikes have been observed, for example, in anticipation of competition and in response to winning (Oliveira et al., 2009; Salvador, 2005). In parallel, testosterone appears to downregulate processes related to social affiliation, for example, by decreasing facial mimicry (Hermans et al., 2006), empathic accuracy (van Honk et al., 2011), and trust (Bos et al., 2010). There is also some preliminary evidence suggesting that listening to various forms of music may lower testosterone levels in males (Fukui, 2001; Fukui and Toyoshima, 2013; Fukui and Yamashita, 2003), which, if extended to music making, may indicate that group singing can act on testosterone alongside oxytocin and cortisol to further modulate affiliation and social bonding.

In sum, group singing is associated with a variety of benefits relevant to health and wellbeing. Among these, social benefits hold both practical and theoretical significance. Here, we investigate the relationship between group singing and endocrine factors that are associated with affiliation and social bonding, focusing on oxytocin, cortisol, and testosterone, assayed in saliva. Previous studies offer conflicting evidence on the association between oxytocin and group singing, but suggest consistent decreases in cortisol after singing in relatively low-stress contexts. For both factors, there is evidence of stronger effects when singing occurs in a group as opposed to alone (Schladt et al., 2017). In contrast, no studies have yet examined whether effects of group singing on endocrine factors also depend specifically on its musical component, or whether they are instead general to any form of coordinated group activity. Nor have any studies examined potential effects of group singing on testosterone. Accordingly, we designed the present study to test variation in the salivary concentrations of oxytocin, cortisol, and testosterone as a function of the musical (singing vs. speaking) and social (together vs. alone) components of group singing. We additionally collected survey data to determine effects of our experimental manipulations on self-perceived affective status and feelings of social connection, and used heart rate monitors to test for differences in physical exertion between conditions.

## 2. Methods

### 2.1. Participants

The participants in this study were 71 members of the *Wiener Jeunesse-Chor* (“Vienna Youth Choir”; 45 female, 26 male). They ranged in age from 17 to 28 years old (*mean* = 23.11, *s.d.* =2.6). This choir is organized around the academic semester. At the start of each semester, returning members join new members under the direction of a professional voice instructor to develop a repertoire of songs that is publicly performed at the semester's end. Our study was conducted at the midpoint of the winter semester, when the current members had been singing together for a minimum of 8 weeks. No choir members were professional singers. All participants provided written informed consent and all experiments were approved by the University of Vienna Ethics Committee (protocol number 00063). Sample size was determined by the number of participants in the choir that volunteered to participate.

### 2.2. Experimental protocol

**Fig. 1.**
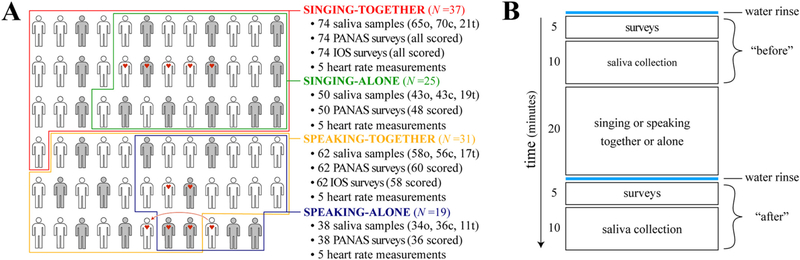
Study sample and experimental procedure. (A) The 71 participants in this study enclosed by colored lines indicating the experimental conditions they took part in (shading indicates sex; males in gray, females in white). Participants enclosed by multiple colors took part in multiple conditions (always in the same vocal mode). Red hearts indicate participants that wore wireless heart rate monitors in the conditions they took part in. The red arrow indicates the one heart rate monitor in the speaking-alone condition that was moved to a different individual in the speaking-together condition; see main text). Bullet points describe the data collected in each condition, including the total number of saliva samples collected and subsets assayed for oxytocin “o”, cortisol “c”, and testosterone “t”, the total numbers of surveys administered (and subsets scored), and the number of heart rate measurements made. (B) The order of events in each experimental condition shown over time.

The experimental manipulations crossed two factors –vocal production mode (singing vs. speaking) and social context (together vs. alone) – resulting in four experimental conditions: (1) singing-together, (2) speaking-together, (3) singing-alone, and (4) speaking-alone (Fig. 1A). All experimental conditions followed the same 50-min protocol (Fig. 1B), with surveys and saliva collection taking place before and after a core 20-min of activity. All experiments took place on Monday evenings from 18:00–20:00, shortly before or during the choir's regularly scheduled practice.

The singing-together and speaking-together conditions took place on the same Monday evening, each in a different large room at the choir's regular meeting place, the *Musisches Zentrum Wien* (“Music Center Vienna”). Sixty-eight of the 71 participants took part in the together conditions. At the beginning of the session, they were pseudo-randomized to the singing-together condition (*n* = 37) or the speaking-together condition (*n* = 31), balancing for sex (14 and 10 males respectively) and vocal range (soprano, alto, tenor, bass) as allowed by our sample (see Fig. 1A). In the singing-together condition, participants stood and sang in chorus for 20 min led by the choir director, including 5-min of vocal warm-up and 15 min of singing songs from the choir's repertoire (excerpts from *The Armed Man* by Karl Jenkins). The director was asked to not interrupt/correct, and to keep pauses as short as possible (30 s or less). In the speaking-together condition, participants stood and spoke the lyrics of the same songs for 20 min led by co-author JG (a member of the choir). Prior to starting, JG asked participants to speak the lyrics like a narrative, without following a particular tune or rhythm. Because speaking song lyrics takes less time than singing them, JG led the speakers through the repertoire ~1.5 times until the allotted 20 min had elapsed.

The singing-alone and speaking-alone conditions took place over six Monday evenings, half scheduled before the Monday of the together conditions and half scheduled afterwards to balance out potential order effects for participants who completed both an alone condition and a together condition (*n* = 41, always in the same vocal mode; see Fig. 1A). The alone conditions were matched to the together conditions as closely as possible, with the exception that participants vocalized alone in acoustically isolated practice rooms (also located at the Music Center Vienna). A total of 44 choir members participated in the alone conditions, pseudo-randomized to the singing-alone condition (*n* = 25) or the speaking-alone condition (*n* = 19), balancing for sex (11 and 7 males respectively) and vocal range as allowed by the sample (see Fig. 1A). The procedures for the singing-alone and speaking-alone conditions were the same as for the singing-together and speaking-together sessions, but participants led themselves through written instructions.

### 2.3. Saliva collection

Saliva samples were collected using a passive drool technique in which the participant sat leaning forward, allowing saliva to flow with gravity through a purpose-built tube into a 3.5 ml cryovial (Stratech Scientific, Ely, UK). Each saliva collection period was 10 min long (see Fig. 1B). To reduce the risk of contaminated samples, participants were asked not to consume meals for at least one hour before each session, and to avoid foods or drinks containing acid, sugar, or caffeine for at least 15 min before each session. Additionally, participants were supplied with water at the sessions and asked to rinse their mouths 5 min prior to the start of each saliva collection period (see Fig. 1B). Immediately after each saliva collection period, the cryovials were placed on dry ice, then transferred 1–2 h later to a freezer, where they were stored at −80 °C until further processing.

### 2.4. Hormone quantification

A total of 224 saliva samples were collected across the four experimental conditions. These samples were thawed and centrifuged at 1660 *g* for 20 min. The supernatant was aliquoted into microcentrifuge tubes and refrozen until analysis with the exception of the aliquots used for oxytocin, which were processed immediately. All standards and samples were run in duplicates for all assays. A small number of samples could not be assayed for all analytes due to insufficient test volume and/or corruption with extraneous material (15 for oxytocin, 16 for cortisol, and 8 for testosterone). In addition we excluded samples with intra-assay coefficients of variation (*cv;* calculated from the concentrations of duplicate aliquots of the same sample) *>* 20% from further analysis (9 for oxytocin, 3 for cortisol, and 10 for testosterone).

Oxytocin concentration was quantified using an enzyme-linked immunosorbent assay (ELISA) kit following the protocol provided by the kit manufacturer (Arbor Assays, Ann Arbor, USA; Catalogue Nr. K048). This kit was selected because of its low-cross reactivity with human neuropeptides closely related to oxytocin, such as arginine vasopressin (cross reactivity =0.12%), but relatively high crossreactivity with mesotocin (=88.4%), an oxytocin-like hormone found in non-mammalian species also studied by our lab. Prior to the ELISA, an extraction procedure was performed: 300 μl of saliva were diluted 1:1.5 with the kit extraction solution, vortexed, and incubated on shaker for 90 min. After 4 °C centrifugation at 1660 *g* for 20 min, the supernatant was transferred into a glass tube and dried down under N_2_ stream at 37 °C. The extracts were then resuspended in 250 μl of assay buffer. Samples from the same individual were always run in the same assay, meaning no within-individual variation is attributable to intra-assay variation. Final concentrations were corrected for the dilution factor. The sensitivity of the assay was 17.0 pg/ml, the inter-assay *cv* of controls was *<*8%, and the mean intra-assay *cv* of duplicates was 4.4% (*s.d.* =4.26%).

Free cortisol assays were conducted following the protocol provided by the ELISA kit manufacturer (Demeditec, Kiel, DEU; Catalogue Nr. DES6611). Aliquots were thawed, vortexed, and centrifuged for 20 min at 1660 *g*. The sensitivity of the assay was 0.024 ng/ml, the inter-assay *cv* of controls was *<*8%, and the mean intra-assay *cv* of duplicates was 4.1% (*s.d.* =3.82%).

Testosterone assays were conducted on samples from male participants only (*n* = 86) following the protocol provided by the kit manufacturer (IBL International, Hamburg, DEU; Catalogue Nr. RE2631). Aliquots were thawed, vortexed, and centrifuged for 10 min at 3000 *g*. The sensitivity of the assay was 10 pg/ml, the inter-assay of controls *cv* was *<*11%, and the mean intra-assay *cv* of duplicates was 4.75% (*s.d.* =4.9%).

We remind the reader that because we measured the above endocrine factors with immunoassays, our concentration values may include other factors that cross-react with the antibodies used for the analyses, e. g., arginine-vasopressin, corticosteroids other than cortisol, and androgens other than testosterone.

### 2.5. Other measures

The German version of the Positive And Negative Affect Survey (PANAS; Krohne et al., 1996) was used to assess self-perceived affective status. The PANAS comprises 20 items, 10 focused on positive affect (e. g., “Active”, “Enthusiastic”, “Strong”, and 10 focused on negative affect (e.g., “Irritable”, “Distressed”, “Nervous”), asking respondents to assess their feelings on each from “not at all” to “very much” (coded 0–4; Watson and Clark, 1988). The PANAS was administered to every participant before and after each experimental manipulation (224 times total) in the five-minute periods immediately preceding saliva sample collection (see Fig. 1B). For each instance of the PANAS, responses on the positive and negative items were separately summed, and the difference (positive minus negative) was calculated to compute a composite “total affect” score. For statistical reasons (see below), we linearly transformed these scores from their original range (−40 to +40) to range between 0 and 1. Scores *>*0.5 indicate that self-perceived affective status is net positive; scores *<*0.5 indicate that self-perceived affective status is net negative. Six PANAS questionnaires were excluded due to incomplete responses, leaving 218 for analysis.

Self-perceived social connectedness was assessed using a modified version of the Inclusion of Other in the Self (IOS) scale (Aron et al., 1992). The IOS scale was administered to every participant in a “together” condition before and after each experimental manipulation (136 times total). Responses were prompted by the question *Welches Diagramm beschreibt am besten wie nahe du dich deinen ChorkollegInnen fühlst?* (“Which diagram best describes how close you feel to your fellow choir members?”). Participants responded by choosing one of seven diagrams labelled 1–7. Each diagram consisted of two circles, one representing the self (“*Ich*”) and one representing the other (“*Andere*”), and they ranged from not overlapping at all (score = 1) to almost fully overlapping (score = 7). Four IOS questionnaires were excluded due to incomplete responses, leaving 132 for analysis.

Physical exertion was assessed using heart rate (Gamberale, 1972), monitored for a subset of participants during experimental manipulations using a Polar Team Pro wireless sensor system (Polar Electro, Kempele, FIN). During together conditions, this system was configured to simultaneously measure the heart rates of 10 participants (5 singers [2 males] and 5 speakers [3 males]) via individual chest-mounted Polar H7 heart rate sensors. The individuals that wore the sensors were selected to roughly match in height and weight between the singing and speaking conditions. During the alone conditions, heart rate was assessed in the same individuals using the same system. One exception was a female participant that had her heart rate assessed in the speaking-alone condition but was absent on the day of the speaking-together condition due to illness. Another female participant of similar height and weight was thus selected to replace her in the speaking-together condition (see Fig. 1A). In all conditions, heart rate was monitored for the entire 20-min experimental manipulation, with the core 10 min of each individual’s data being used to calculate a mean value in beats per minute (bpm).

### 2.6. Statistical analysis

A separate Linear Mixed Model (LMM) was used to estimate the effects of our experimental conditions on the salivary concentrations of each of the three hormones. The full version of each model included fixed effects for time (before or after experimental manipulation), vocal mode (singing or speaking), social context (together or alone), and their three-way interaction as predictors. Test order (first or second for participants who completed two conditions; first for participants that only completed one), and sex (male or female; excluded in the testosterone model) were additionally included as control predictors to account for their potential influence on hormone levels, and a random intercept effect was included to account for variance attributable to individual differences between participants. In this modeling framework, effects of our experimental manipulations on hormone levels are registered by interactions between time and vocal mode, time and social context, or the three-way interaction between time, vocal mode, and social context. Main effects of vocal mode, social context, and their two-way interaction do not address experimental effects on salivary hormone levels because they confound samples taken before and after our experimental manipulations. The number of observations (i.e., hormone concentration measurements) per estimated effect was 16.67 for the oxytocin model and 17.08 for the cortisol model, but just 6.18 for testosterone model, indicating low power.

Prior to fitting each model, we inspected the response variables and consequently logarithmically transformed (base-*e*) the cortisol and oxytocin levels to achieve more symmetric distributions. After fitting each model, we visually inspected QQ-plots of the residuals and fitted versus residuals plots to check that LMM assumptions of normally distributed and homogenous residuals were satisfied (Field, 2005; Quinn and Keough, 2002); no deviations were indicated in either case for any model. Collinearity of the predictors was assessed for each model (specified without interactions and without the random effect for individual) by examining maximum variance inflation factors (*vif*). No issues with collinearity were found (*vif* = 1.051 for oxytocin, 1.058 for cortisol, 1.026 for testosterone; Quinn and Keough, 2002). Finally, the stability of each model was assessed by iteratively excluding one level of the random effect at a time (i.e. dropping one individual from the analysis) and examining changes in estimated coefficients (Nieuwenhuis et al., 2012). Stability was acceptable for the oxytocin and cortisol models (see “max” and “min” columns in Tables 1–2). This assessment was not made for the testosterone model (see Results).

After model diagnostics, each “full model” was compared to a parallel “null model” to determine if the effect structure of the full model improved prediction of salivary hormone concentrations to a significant degree (Forstmeier and Schielzeth, 2011; Mundry and Nunn, 2009). The null models included time, sex, test order, and the random intercept effect, but lacked predictors representing the experimental manipulations (i.e., vocal mode and social context). Only when a full versus null model comparison indicated a significant difference, did we proceed with post-hoc significance testing of individual fixed effects. Post-hoc tests were made using likelihood ratio tests to compare full models to reduced models dropping one fixed effect at a time (Barr et al., 2013; Dobson, 2002). If the three-way interaction (time × vocal mode × social context) was determined to be non-significant during this process, a reduced model was fit replacing the three-way interaction with two two-way interactions (time × vocal mode, and time × social context) and the “drop one” significance testing procedure was repeated. If either two-way interaction was found non-significant, another reduced model was fit excluding it as well. Finally, the significance of post-hoc tests for each model was determined by comparison with a family-wise α-level of 0.05, corrected for multiple comparisons using the Holm-Bonferroni method (Holm, 1979).

The effects of our experiment on self-perceived affective status, as indicated by PANAS total affect scores, were estimated using a Generalized Linear Mixed Model (GLMM) with a beta error distribution and a logit link function to account for the fact that PANAS scores are bounded (Bolker, 2008; using a beta distribution additionally requires that the data fall between 0 and 1). In parallel to the hormone models, the full PANAS model included fixed effects for time, vocal mode, social context, their three-way interaction, test order, and sex, as well as a random intercept effect for individual. Model diagnostics indicated that the full PANAS model did not suffer from issues with overdispersion (dispersion parameter =0.814), collinearity (maximum *vif* = 1.049), or stability (see “max” and “min” in Table 3). Full versus null model comparisons and post-hoc tests were conducted for the PANAS model in the same fashion as described above for the hormone models. The number of observations per estimated effect for the PANAS model was 16.77.

The effects of our experiment on self-perceived social connectedness, as indicated by the IOS scale, were estimated using a Cumulative Link Mixed Model (CLMM) with a logit link function to account for the fact that IOS scale data is discrete and ordinal (Christensen and Brockhoff, 2013). This model predicts the cumulative logarithm of the odds of receiving an IOS score of *j* or lower, as a function of our experimental manipulations. By reversing the logit transformation on model estimates (exp[*x*]/[1 + exp.(*x*)], where *x* = model estimate), we derived the cumulative probability of each IOS response, and by subtracting the cumulative probability of scores below *j* (i.e. *j*-1), we derived the non-cumulative probability for each of the seven IOS scale responses. In Fig. 6B, model results are shown as weighted averages, obtained by multiplying the non-cumulative probability of each IOS scale response with the respective level of its score and summing them up. The effect structure of the full IOS model was parallel to those of the full hormone and PANAS models, with the exception that social context and its interactions were not included because the IOS scale was only administered in the group conditions. The full IOS model thus included fixed effects for time, vocal mode, their two-way interaction, test order, and sex, as well as a random intercept effect for individual. Model diagnostics indicated that the full IOS model did not suffer from issues with collinearity (maximum *vif* = 1.016) or stability (see “max” and “min” in Table 4). Full versus null comparisons and post-hoc tests were performed in the same fashion as described above. The number of observations per estimated effect for the IOS model was 11.

Finally, the effects of our experiment on physical exertion as indicated by mean heart rate measurements were estimated using an LMM. The full heart rate model was designed to predict mean bpm as a function of fixed effects for vocal mode, social context, and their two-way interaction, as well as a random effect for individual. Time was not included because heart rate measurements were made during experimental manipulations rather than before and after them. Test order and sex were excluded because the number of observations per estimated effect was already quite small (3.33) and these were the only effects that could be excluded while still addressing our experimental manipulations. Model diagnostics and significance testing were performed in the same fashion as described above (maximum *vif* = 1.016; see Table 5 for stability).

All models were fit in R (version 3.5.3) using the function ‘lmer’ from package ‘lme4’ (version 1.1–21; Bates et al., 2015) for the hormone and heart rate models, the function ‘glmmTMB’ from package ‘glmmTMB’ (version 0.2.3; Brooks et al., 2017) for the PANAS model, and the function ‘clmm’ from the package ‘ordinal’ for the IOS model (version 2019.12–10; Christensen, 2019). VIFs were determined using the function ‘vif’ from package ‘car’ (version 3.0–3; Fox and Weisberg, 2011). Assessments of model stability were made using custom functions kindly provided by Dr. Roger Mundry (as described above). Confidence intervals in the hormone and heart rate models were calculated using the function ‘bootMer’ from package ‘lme4’ to conduct parametric bootstrap analyses (1000 iterations). Confidence intervals in the PANAS model were calculated using the function ‘simulate’ from package ‘glmmTMB’. Confidence intervals in the IOS model were calculated using a nonparametric bootstrap analysis (1000 iterations), randomly sampling individuals from the original data set (with replacement) and fitting a new model at each iteration. Likelihood ratio tests were performed using the function ‘anova’ from package ‘stats’ (R Core Team, 2012). For plotting and reporting significant effects, the estimates for each of the other predictors were centered by replacing them with their mean value calculated across levels, weighted by the proportions of observations in each level.

## 3. Results

### 3.1. Hormone assays

**Fig. 2.**
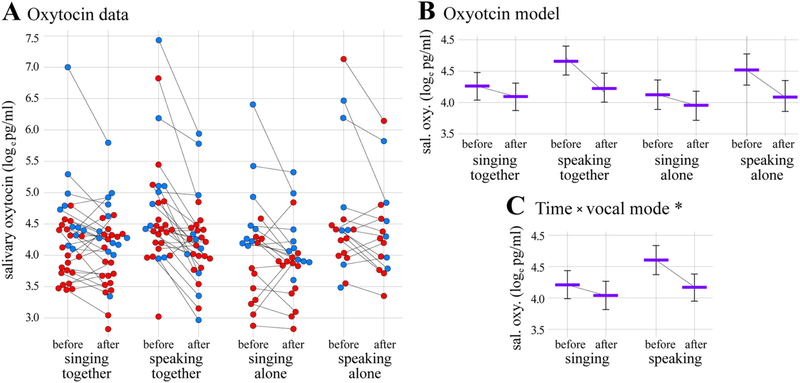
Oxytocin results. (A) Log-transformed salivary oxytocin concentrations plotted as a function of time, vocal mode, and social context. Each circle represents one sample, red = female, and blue = male. Diagonal black lines connect samples from the same participant. (B) Results of the oxytocin linear mixed model analysis showing estimated average salivary oxytocin concentrations as a function of time, vocal mode, and social context, drawn with estimated effects for test order and sex centered. (C) Model results showing the significant two-way interaction between time and vocal mode (*p = 0.018), drawn with other estimated effects centered. Error bars in B & C indicate 95% confidence intervals.

An overview of the hormone data is provided in Table 1 and the full data set is provided in Supplementary Data 1. The results of the oxytocin analysis are shown in Fig. 2 and Table 2. The data comprised 200 measurements (186 paired, 14 unpaired) from 68 individuals (44 female, 24 male). The full oxytocin model was a significantly better predictor of salivary oxytocin concentrations than the null model, *χ^2^* = 16.43, *d.f.* =6, *p* = 0.0116. Post-hoc tests indicated that neither the three-way interaction between time, vocal mode, and social context, nor the two-way interaction between time and social context, were significant (*p* = 0.190 and *p* = 0.230 respectively). However, the two-way interaction between time and vocal mode was significant (*estimate* ± *s. e.* =0.265 ± 0.111; *χ^2^* = 5.7, *d.f.* =1, *p* = 0.018). Specifically, salivary oxytocin concentrations decreased after both singing and speaking, but the decrease after singing was estimated to be much smaller than the decrease after speaking (− 10.4 pg/ml vs. −35.1 pg/ml respectively; Fig. 2C). The effect of the test order control predictor was not significant (Table 2), but the effect of the control predictor sex was significant (*estimate* ± *s.e.* =0.51 ± 0.15; *χ^2^* = 10.22, *d.f.* =1, *p* = 0.001). Specifically, salivary oxytocin concentrations were estimated to be higher in males (96.1 pg/ml [*c.i.* =75.1–122.1]) compared to females (57.4 pg/ml [47.3–68.7]). Sex did not interact with the time × vocal mode interaction (i.e., testing the three-way interaction between sex, time, and vocal mode showed that it was not significant, *χ^2^* = 0.116, *d.f.* =1, *p* = 0.733). Thus, salivary oxytocin concentrations were found to decrease less after singing than after speaking, independent of participant sex.

**Fig. 3.**
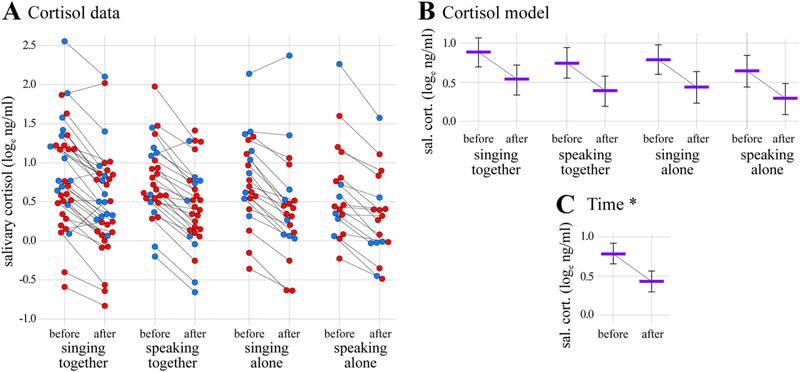
Cortisol results. (A) Log-transformed salivary cortisol concentrations plotted as a function of time, vocal mode, and social context. (B) Results of the cortisol linear mixed model analysis showing estimated average cortisol levels as a function of time, vocal mode, and social context, drawn with estimated effects for test order and sex centered. (C) Model results showing the significant main effect of time (*p < 0.001), drawn with other estimated effects centered. Format is the same as in Fig. 2.

The results of the salivary cortisol analysis are shown in Fig. 3 and Table 3. The data comprised 205 measurements (202 paired, 3 unpaired) from 69 individuals (45 females, 24 males). The full cortisol model was a significantly better predictor of salivary cortisol concentrations than the null model, *χ^2^* = 13.15, *d.f.* =6, *p* = 0.041. Post-hoc tests indicated that three-way and two-way interactions between time, vocal mode, and/or social context were not significant (*ps* ≥ 0.782). The effects of the control predictors test order and sex were similarly not significant (Table 3). Instead, the only significant predictor in the cortisol model was the main effect of time (*estimate* ± *s.e*. = − 0.35 ± 0.04; *χ^2^* = 65.03, *d.f.* =1, *p <* 0.001). Specifically, salivary cortisol was estimated to be higher before the experiments compared to after the experiments, regardless of condition (2.18 ng/ml [*c.i.* =1.93–2.51] vs. 1.54 ng/ml [1.35–1.76) respectively; Fig. 3C). Thus, salivary cortisol concentrations were found to decrease from before to after the experiments, but not in a way that was specific to the experimental manipulations of vocal mode or social context.

**Fig. 4.**
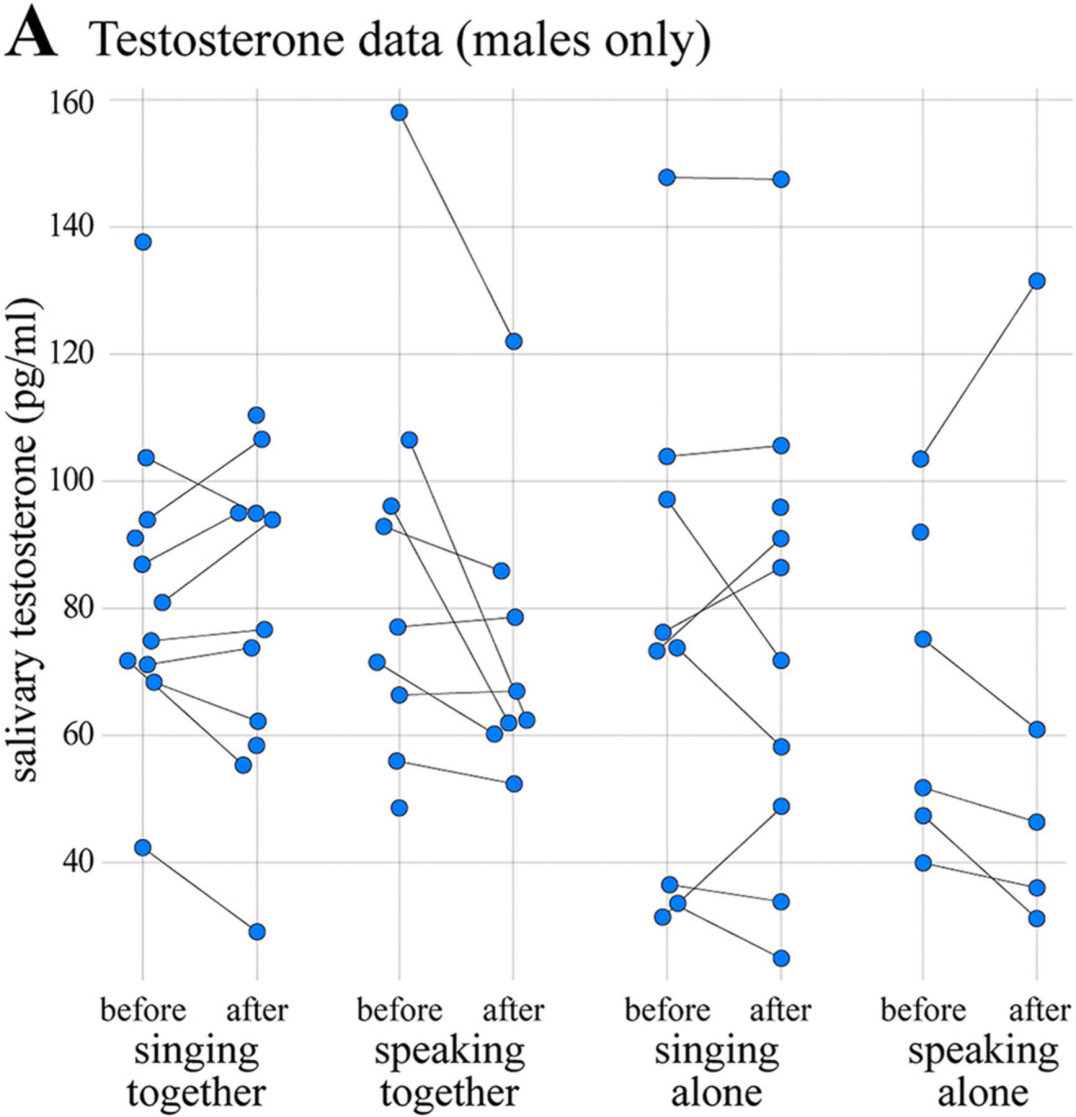
Testosterone results. Salivary testosterone concentrations in males plotted as a function of time, vocal mode, and social context. Format is the same as in Figs. 2 & 3.

The results of the salivary testosterone analysis are shown in Fig. 4. The data comprised 68 measurements (62 paired, 6 unpaired) from 22 individuals (all male). In contrast with the oxytocin and cortisol results, the full testosterone model was not a significantly better predictor of salivary testosterone concentrations than the null model, *χ^2^* = 6.75, *d.f.* =6, *p* = 0.345. This implies that the experimental manipulations did not have significant effects on male salivary testosterone concentrations in this study. No post-hoc tests were therefore administered and the analysis was terminated.

### 3.2. Other measures

**Fig. 5.**
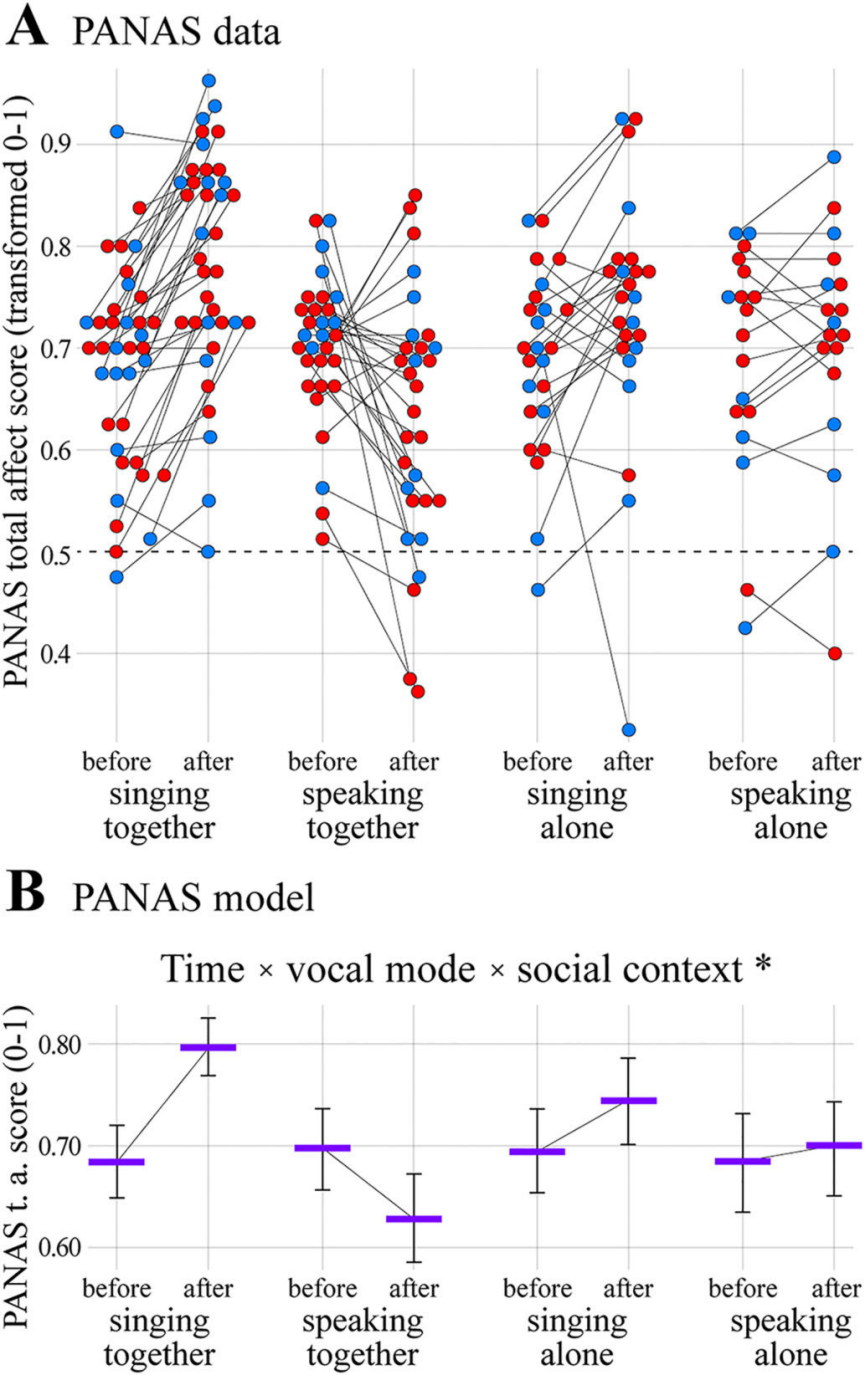
Affect results. (A) Positive And Negative Affect Schedule (PANAS) total affect scores transformed to range between 0 and 1 plotted as a function of time, vocal mode, and social context. Scores above the horizonal dashed line indicate positive affect *>* negative affect, scores below the dashed line indicate positive affect *<* negative affect. (B) Results of the PANAS generalized linear mixed model analysis showing estimated average transformed PANAS total affect scores as a function of time, vocal mode, and social context (and their significant three-way interaction; *p = 0.001), drawn with estimated effects for test order and sex centered. Format is the same as in Figs. 2–4.

The results of the affect analysis are shown in Fig. 5 and Table 4. The data comprised 218 scored questionnaires (all paired) from 70 individuals (44 females, 26 males). The full PANAS model was a significantly better predictor of PANAS total affect scores (transformed to range between 0 and 1) than the null model, *χ^2^* = 50.77, *d.f.* =6, *p <* 0.0001. Post-hoc tests indicated that the three-way interaction between time, vocal mode, and social context was significant (*estimate* ± *s.e.* =0.73 ± 0.22; *χ^2^* = 10.54, *d.f.* =1, *p* = 0.001). Specifically, transformed PANAS total affect scores were estimated to increase by 0.11 after singing-together, 0.05 after singing-alone, and 0.02 after speaking-alone, but decrease by 0.07 after speaking-together (+9.0, +3.8, +1.2, and − 5.6 untransformed PANAS points respectively; Fig. 5B). No significant effects of the control predictors test order or sex were observed (Table 4). Thus, vocal mode and social context were found to interact in shaping effects on self-perceived affective status, with singing together resulting in the greatest benefit.

**Fig. 6.**
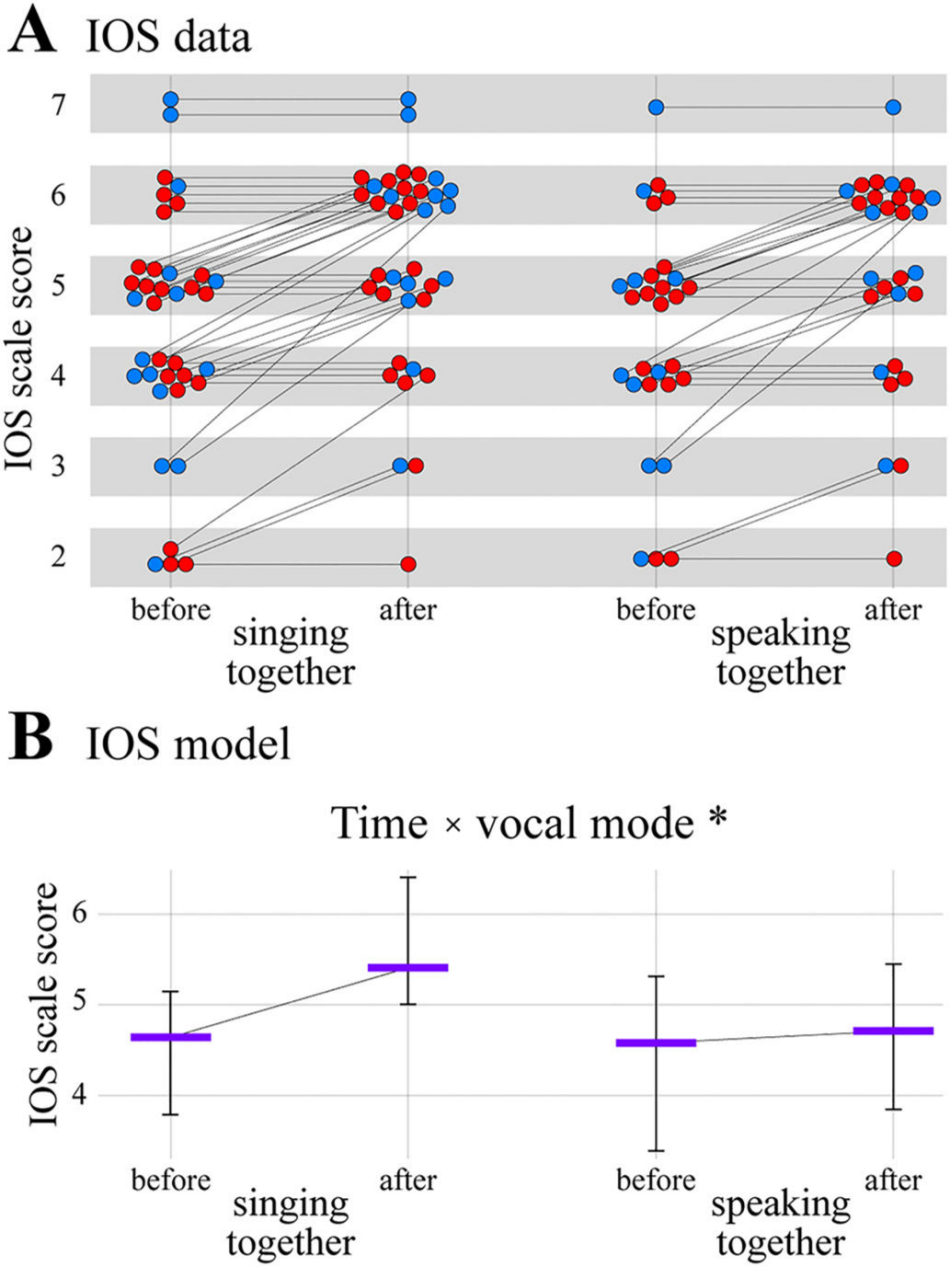
Social connectedness results. (A) Inclusion of the Other in the Self (IOS) scale responses shown as a function of time and vocal mode. (B) Results of the IOS cumulative link mixed model analysis showing estimated average IOS scale responses as a function of time and vocal mode (and their significant two-way interaction, *p = 0.008), drawn with estimated effects for test order and sex centered. Format is the same as in Figs. 2–5.

The results of the social connectedness analysis are shown in Fig. 6 and Table 5. The data comprised a total of 132 scored surveys (all paired) from 66 individuals (42 females, 24 males). The full IOS model was a significantly better predictor of IOS ratings than the null model, *χ^2^* = 8.771, *d.f.* =2, *p* = 0.012. Post-hoc tests indicated that the two-way interaction between time and vocal mode was significant (*estimate* ± *s. e.* =1.95 ± 0.75; *χ^2^* = 7.06, *d.f.* =1, *p* = 0.008). Specifically, IOS scale responses were estimated to increase more after singing together (+0.77) than after speaking together (+0.13; Fig. 6B). Note that the IOS model did not include social context as a predictor because it was only administered in together conditions. The effect of the control predictor sex was not significant (Table 5), but the effect of the control predictor test order was significant (*estimate* ± *s.e.* =2.20 ± 0.91; *χ^2^* = 6.16, *d.f.* =1, *p* = 0.013). Specifically, IOS scale responses were estimated to be higher in second sessions (5.10 [*c.i.* =4.51–5.99]) compared to first sessions (4.30 [3.56–5.07]). Test order did not interact with the time × vocal mode interaction (i.e., testing the three-way interaction between test order, time, and vocal mode showed that it was not significant, *χ^2^* = 0.286, *d.f.* =1, *p* = 0.593). Thus, singing together was found to increase feelings of social connectedness more than speaking together, regardless of the order in which these conditions took place.

**Fig. 7.**
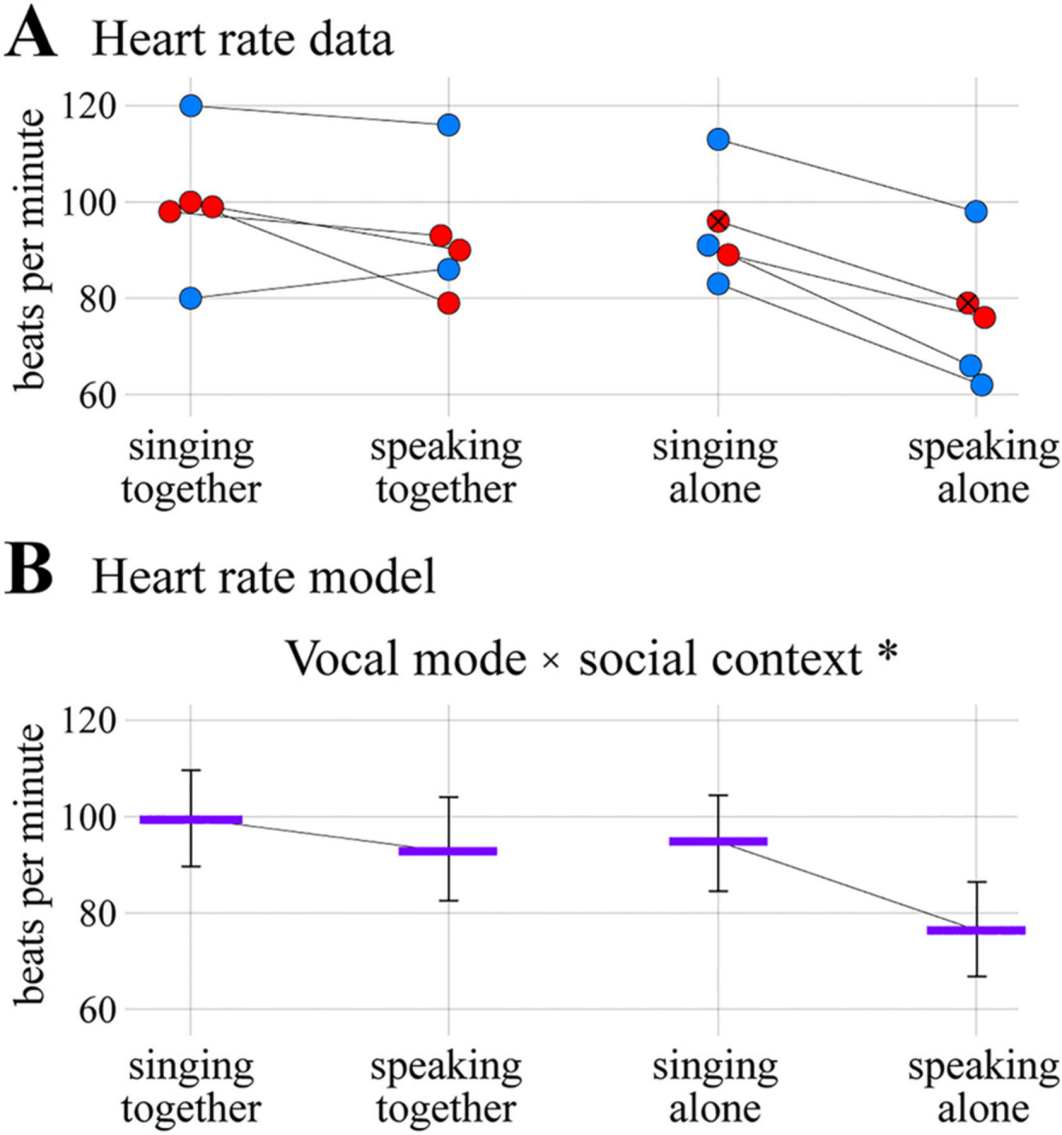
Physical exertion. (A) Heart rate in beats per minute shown as a function of vocal mode and social context. Each circle represents a mean heart rate value for one participant calculated over the core 10 min of one experimental condition. Diagonal black lines connect measurements from the same individual, with the exception of the line between the two circles marked with Xs, which came from different individuals (see main text). (B) Results of the heart rate linear mixed model analysis showing the significant interaction between vocal mode and social context (*p = 0.029). Format is the same as in Figs. 2–6.

The results of the physical exertion analysis are shown in Fig. 7 and Table 6. The full heart rate model was a significantly better predictor of mean heart rate than the null model, *χ^2^* = 16.534, *d.f.* =3, *p <* 0.0001. Post-hoc tests indicated a significant two-way interaction between vocal mode and social context (*estimate* ± *s.e.* = − 11.83 ± 4.75; *χ^2^* = 4.79, *d.f.* =1, *p* = 0.029). Specifically, heart rate was estimated to be highest in the singing-together condition (99.4 bpm [*c.i.* =89.8–109.8]), followed closely by the speaking-together and singing-alone conditions (94.8 [84.5–104.4], and 92.8 [82.5–104.0] respectively), and lowest in the speaking-alone condition (76.2 [66.9–86.4]). Note that the heart rate model did not include time, test order, or sex as predictors. Thus, heart rate was found to be highest during singing together, but similar in all other conditions except for speaking alone, where it was considerably lower.

### 3.3. Analysis of correlations

Finally, we examined the relationships between each pair of dependent variables measured in this study (see correlation matrix in Supplementary Fig. 1A). Although we did not find any significant relationships between salivary hormones concentrations and any of the other variables that we measured, we did find significant positive correlations between salivary oxytocin and cortisol (*Pearson's r* = 0.27, *p <* 0.001), salivary cortisol and testosterone (*r* = 0.33, *p* = 0.0124), and PANAS total affect scores and IOS scale responses (*r* = 0.36, *p <* 0.0001). Examining these relationships in greater detail, it was apparent that the oxytocin-cortisol correlation was primarily driven by measurements made in the singing-together condition (*r* = 0.43, *p* = 0.0005), as correlations in the other conditions were very weak and/or nonsignificant (Supplementary Fig. 1A). Likewise, the cortisol-testosterone relationship was primarily driven by measurements made in the speaking conditions (*r* = 0.77, *p* = 0.0007 for speaking-together; *r* = 0.75, *p* = 0.0117 for speaking-alone), as correlations in the singing conditions were nonsignificant. By contrast, the PANAS-IOS relationship was more comparable between the singing and speaking (*r* = 0.37, *p* = 0.0130 for singing-together; *r* = 0.28, *p* = 0.0321 for speaking-together; IOS not administered in alone conditions). We next examined relationships between the changes that occurred in each pair of dependent variables from before to after our experimental manipulations (see correlation matrix in Supplementary Fig. 1B). Here, the only significant correlation was between changes in PANAS total affect scores and IOS scale responses (*r* = 0.59, *p <* 0.0001). This relationship was significant in both the singing-together (*r* = 0.42, *p* = 0.0098) and speaking-together (*r* = 0.57, *p* = 0.0013) conditions.

### 4. Discussion

The hormone results described here indicate overall decreases in salivary concentrations of oxytocin and cortisol, and an absence of significant effects on salivary concentrations of testosterone in males. With respect to our experimental manipulations, vocal mode was determined to affect salivary oxytocin, with decreases being smaller after singing compared to speaking. In parallel, the survey results indicated that vocal mode and social context interacted to affect self-perceived affective status, with singing together producing the largest positive shift (see also Kreutz, 2014; Pearce et al., 2015; Schladt et al., 2017; Weinstein et al., 2016). Singing together was additionally found to be more effective than speaking together at stimulating feelings of social connectedness (see also Pearce et al., 2015; Weinstein et al., 2016). Finally, as might be expected, heart rates measured for ten participants suggested that physical exertion was highest during singing together and lowest during speaking alone. We now discuss each hormone result in turn (order-reversed from above), adding context from prior literature, exploring potential interpretations, and discussing connections with our other measures. We conclude by considering the connection between music and social bonding, as well as implications for future studies on the endocrinological correlates of musical behavior.

### 4.1. Testosterone

The absence of any significant effect on concentrations of testosterone in male saliva indicates that our experimental manipulations of vocal mode and social context were insufficient to systematically influence the acute regulation of testosterone in males. This is an important preliminary finding, but we emphasize caution in its interpreted because of the relatively low power of our testosterone model (observations per estimated effect =6.18). Nevertheless, we saw no indication that potential acute effects of music listening on male testosterone levels extend to singing (Fukui, 2001; Fukui and Toyoshima, 2013; Fukui and Yamashita, 2003). Future studies examining testosterone in the context of group singing or other musical behaviors should consider incorporating further factors or measurements potentially relevant to testosterone, such as the existence of interpersonal friendships, individual social status, singing skill, and competitive/cooperative group dynamics (Casto and Edwards, 2016; Edwards et al., 2006; Ponzi et al., 2016). It may also be useful to study testosterone and group singing over longer periods of time, as changes may appear gradually as participants move from forming new social bonds towards maintaining existing ones (Kornienko et al., 2016). Finally, it will be important for future studies to examine testosterone levels in females (Grant and France, 2001; van Anders, 2013), especially given that most modern choirs are majority female (Elpus, 2015).

### 4.2. Cortisol

The experimental manipulations did not differentially impact concentrations of cortisol in saliva, which decreased markedly from before to after participation regardless of condition. Whether the magnitude of the decrease that we observed was specifically related to our experiment, as opposed to natural diurnal cycling, cannot be determined with certainty. A recent meta-analysis that aggregated cortisol data from more than 18,000 individuals found an average diurnal decrease during the time at which our experiment took place of approximately 12% per 30 min (range: 3% to 20% assuming wake-times from 7:00 and 10:00; Miller et al., 2016). On the basis of this data, it may be argued that the magnitude of the cortisol decrease that we observed (30%) was too large to be explained by diurnal cycling alone, which would in turn suggest that recreational singing or speaking, together or alone, comprise particularly effective ways to relax. That said, we emphasize that testing this hypothesis would have required inclusion of a “no-treatment” control condition in which hormone levels were assessed at the same times but in the absence of any experimental manipulations. Although such control conditions are not typically included in non-clinical research, it is important that future studies aiming to identify experimental effects of group singing on cortisol levels prioritize their inclusion, particularly if measurements are made in the evening, as they were here, and as they have been in previous studies (Beck et al., 2000; Fancourt et al., 2015, 2016; Kreutz et al., 2004; Schladt et al., 2017).

With respect to previous studies, our cortisol results mostly conformed to expectations. In relatively low-stress recreational contexts like that examined here, four previous studies have reported significant cortisol decreases after group singing (ranging in magnitude from approximately 18% to 27%; Beck et al., 2000; Fancourt et al., 2015, 2016; Schladt et al., 2017), and two others have reported non-significant changes (in opposite directions; Kreutz et al., 2004; Kreutz, 2014). Although all of these studies have been conducted in the evening, some have nonetheless found experimental effects on salivary cortisol. Of particular relevance here, Schladt et al. also compared singing together with singing alone. In contrast with our results, which indicated similar decreases in salivary cortisol after singing together (29%) and singing alone (31%), Schladt et al. found a significantly greater decrease after singing together (~32%^2^) than after singing alone (~20%). This difference in the effects of singing alone between our study and Schladt et al. is particularly notable given that the relevant conditions were highly similar in terms of design and execution (see below). Together, these findings indicate that recreational group singing is typically associated with reduced salivary cortisol concentrations, but that the effects of singing alone are more variable.

### 4.3. Oxytocin

Focusing on group singing first, salivary oxytocin concentrations decreased from before to after participation in our singing-together condition (Fig. 2B). Interpreting the generality of this effect is complicated by the literature. Four previous studies have assessed changes in oxytocin from before to after group singing, all in relatively low-stress contexts. Two of these studies report decreases similar in magnitude to that found here (Fancourt et al., 2016; Schladt et al., 2017), and two report increases (Keeler et al., 2015; Kreutz, 2014). Contrasting these “decrease studies” and “increase studies” provides insight into contextual factors that may modulate the relationship between group singing and oxytocin. The decrease studies include the present one (15% reduction after a 20-min choir rehearsal, *n* = 37), Schladt et al. (~22% reduction after a 20-min choir rehearsal, *n* = 38), and Fancourt et al. (24% reduction after a 70-min choir rehearsal, *n* = 193, across 5 choirs). The increase studies include Kreutz (39% increase after a 30-min choir rehearsal, *n* = 21) and Keeler et al. (~19% increase after a 6 min bout of improvised singing in a jazz quartet, *n* = 4). An additional study, Grape et al. (2003), is also relevant despite not measuring group singing per se (25% increase after a 45-min private singing lesson, *n* = 16). We consider each of the increase studies in detail below, attempting to determine factors underlying their differential effects, relative to our study and other decrease studies.

Starting with the study most different from our own, Grape et al. examined singing lessons between adult students and a teacher with whom they had practiced for a minimum of 6 months. This context differs from all other studies considered here, not only because participants sang alone rather than in a group, but because private lessons involve more focused social interaction than choir rehearsals. This point is particularly important because the intimacy of an interaction appears to be of greater relevance for understanding oxytocin responses to social context than whether an activity is technically performed alone or together with others (Bartz et al., 2011). Additional support for this idea comes from Keeler et al.'s study of improvised singing in a jazz quartet. Although their sample of just four individuals was too small to appropriately evaluate statistical significance, the average oxytocin increase that they observed (~19%) appeared specific to improvised singing—a separate condition where the quartet sang a precomposed jazz standard was instead associated with a small oxytocin decrease (~3%). Taken together, these results suggest that focused collaborative interaction, characteristic of teaching and social improvisation, is likely an important determinant of oxytocin activity in the context of group singing.^3^

The last increase study—Kreutz (2014)—is also the most similar to our own (and the other decrease studies). There are, however, a number of potentially important differences. A first difference is that whereas we and others have examined choirs that existed prior to the initiation of study, Kreutz examined a choir that was specifically formed for their study. Whereas many of the members of our choir had pre-existing friendships and previous experience singing together (often over multiple semesters), the members of Kreutz’s choir presumably had little such relations. This difference in social context is directly relevant to the value of affiliative behavior and potential for social bonding. A second difference is that the choir in our study was made-up entirely of members of the same age cohort (*mean* = 22 years, *range* = 17–28), whereas the choir in Kreutz included a greater mix of ages (*mean* ~ 49, *range* ~ 18–65). Schladt et al.'s choir was similar to ours in participant age (*mean* = 23 years, *range* = 19–29); Fancourt et al.'s was older (*mean* = 59 years) but still relatively narrow in age range (*SD* ~ 12). Given that extrafamilial social bonds are biased towards members of the same age group (Nahemow and Lawton, 1975), the difference in age diversity of the choir studied by Kreutz may have similarly led to differences in affiliative value and bonding potential. Kreutz (2014) is also unique for its connection to a local television station, which ran advertisements to recruit participants, filmed rehearsals for a short documentary feature, and televised a final concert. This televised aspect may have led to a systemic difference in the personality traits of participants, potentially favoring group-level differences in individual oxytocin biology (Li et al., 2015). Finally, the music sung by Kreutz’s choir (e.g., the 1960s pop song “California Dreaming”) was more popular than the relatively obscure choral music used in our study and other decrease studies. Effects of music type on changes in salivary oxytocin concentrations after listening have been reported, and may be attributable to acoustic parameters conveying different types of affect (Ooishi et al., 2017).

A summary of these findings is that group singing in the context of choir rehearsals is primarily associated with decreases in salivary oxytocin concentrations, but that this effect can be modulated to the point of reversal by a variety of factors related to the broader social context in which singing occurs. The intimacy of the social interactions that take place during singing appears to be among the most important of these factors, with more intimate, direct, collaborative interactions potentially driving higher oxytocin activity than more anonymous, diffuse, independent interactions. Related to this, the nature of existing social relationships between group members also appears important, as it sets the stage for differences in the relative value of affiliative behavior and potential for social bonding. Finally, cultural and affective connotations of the specific music being sung may contribute further to differences in the effects of group singing on oxytocin.

Turning to our experimental manipulation of musical versus non-musical vocal production, we found evidence of a smaller decrease in salivary oxytocin concentration after speaking compared to singing (35% vs. 15% respectively; Fig. 2C). This novel comparison suggests that singing may sustain relatively higher levels of oxytocin activity than speaking, a similar vocal behavior that is also used for communication and that also involves high levels of interpersonal coordination. Psychologically, vocal production mode also influenced self-perceived affective status, with singing stimulating greater positive shifts in affect, particularly when performed together with others, as well as greater feelings of social connectedness. What is the basis for these differences in the biological and psychological effects of singing and speaking?

Despite broad similarities, singing and speaking are obviously characterized by a variety of neural, behavioral, and acoustic differences, any of which could be investigated as a potential basis for the differential effects that we observed. One straight-forward approach to understanding the different of effects singing versus speaking on salivary oxytocin is focused on potential differences in physical activity between them. Physical exertion can increase salivary oxytocin concentrations as well as feelings of affiliation (Anshel and Kipper, 1988; de Jong et al., 2015; Tarr et al., 2015). Just 10 min of jogging, for example, was found to increase salivary oxytocin by ~320% on average (de Jong et al., 2015). Accordingly, singing may increase salivary oxytocin concentrations more than speaking because it requires higher levels of physical exertion. Albeit preliminary, our heart rate data do not generally support this possibility. The largest and smallest estimated changes in salivary oxytocin occurred in conditions with approximately equal mean heart rates: salivary oxytocin decreased by 42% after speaking together, where mean heart rate was 94.8 bpm (*SD* = 11.3), but only decreased by 15% after singing alone, despite a similar mean heart rate of 92.8 bpm (*SD* = 14.0; cf. Figs. 2B and 7B). Nevertheless, further experiments are required to fully rule out physical exertion as a potential driver of differential effects of singing and speaking on salivary oxytocin.

Another approach to explaining the observed differential effects is focused on oxytocin's role in mitigating the effects of HPA axis activation (Gibbs, 1986; Neumann, 2002; Schladt et al., 2017). The idea here is that oxytocin activity is upregulated in response to stress, being released alongside cortisol and acting as an anxiolytic (Brown et al., 2016). Accordingly, if singing stimulated greater HPA axis activity than speaking, proportional oxytocin activity may underlie the differential effects that we observed. This interpretation is not supported by our salivary cortisol results, which showed a stable decrease in concentrations across conditions rather than an increase (or smaller decrease) after singing. This suggests that the singing and speaking conditions had similar effects on HPA axis activation. Furthermore, although salivary oxytocin and cortisol concentrations were significantly correlated across all of our measurements, we did not find evidence that these factors changed together over the course of our study. It is therefore unlikely that differential effects of singing versus speaking on salivary oxytocin are explained by differences in activation of the HPA-axis between conditions.

A final issue to consider in accounting for the differential effects of singing versus speaking on salivary oxytocin is focused on baseline differences. Careful inspection of Fig. 2 shows that salivary oxytocin concentrations measured before the speaking conditions were relatively elevated compared to those measured before the singing conditions. Thus, the interaction between time and vocal mode that we observed is confounded with baseline differences in salivary oxytocin between conditions. To test whether or not baseline differences (rather than experimental effects) are responsible for the observed interaction, we fit an alternate oxytocin model that incorporated baseline salivary oxytocin concentrations as a covariate in predicting post-experiment salivary oxytocin concentrations. This “baseline” oxytocin model was a significantly better predictor of post-experiment oxytocin concentrations than a parallel null model (*χ^2^* = 7.85, *d.f.* =3, *p* = 0.049), and post-hoc tests indicated that the two-way interaction between vocal mode and social context was significant (*estimate* ± *s.e.* =0.38 ± 0.16; *χ^2^* = 98.7, *d.f.* =1, *p* = 0.016). Specifically, oxytocin concentrations were estimated to be significantly higher after singing together compared to speaking together (72.7 pg/ml [*c.i.* =64.1–83.1] vs. 56.0 pg/ml [48.4–64.9], *t* (100.5) = −2.497, *p* = 0.014), but not after singing alone compared to speaking alone (62.8 [52.5–74.5] vs. 71.0 [59.2–85.6], *t*(100.5) =0.919, *p* = 0.360). Thus, post-experiment salivary oxytocin concentrations were found to differ as a function of vocal mode after specifically accounting for the influence of baseline differences. This reflects marked differences in the magnitudes of salivary oxytocin decreases across conditions in our original model, and indicates that the corresponding experimental effect is not an artifact of unexplained differences in baseline salivary oxytocin concentrations (see Supplementary Text 1 for further details).

Last, we come to our manipulation of social context. Whereas our original oxytocin model indicates that the effect of vocal mode on salivary oxytocin was not impacted by social context (Fig. 2B), the baseline oxytocin model just described indicates that post-experiment salivary oxytocin concentrations were affected by social context, which interacted with vocal mode. In fact, the pattern of results for singing versus speaking performed together was reversed for singing and speaking performed alone (although the difference was not significant). Nevertheless, in both models, singing together was similarly associated with relatively higher levels of oxytocin activity than speaking together. This is reflected by smaller decreases in salivary oxytocin concentrations from before to after singing versus speaking together in our original model, and higher salivary oxytocin concentrations after singing versus speaking together when controlling for baseline differences in the baseline model. Insight into the disruption of this pattern for singing versus speaking alone in the baseline model can be found by comparing our results with Schladt et al. (2017), who also examined singing together versus singing alone. Although Schladt et al.'s results were similar to ours for singing together (we found an average decrease of 15%, compared to their average decrease of ~22%), their results were markedly different for singing alone (we again found an average decrease of 15%, but they found an average increase of ~10%, Fig. 2B). As noted above in our discussion of discrepant cortisol results between alone conditions in our study and Schladt et al., this suggests that the endocrine effects of singing together are more stable than those of singing alone. This appears to be true despite a high level of procedural similarity between the alone conditions in our study and Schladt et al. In both studies, amateur young-adult chorists were provided with written instructions leading them through a short vocal warm-up followed by practice of their choir's repertoire, for a total duration of 20 min. Additionally, both studies found that singing alone produced positive shifts in self-perceived affect. Hormone changes in response to singing alone thus appear quite sensitive to more subtle differences between individuals and/or contexts (e.g., differences in stress and anxiety, or external performance pressures). Conversely, singing together with others seems to be capable of overriding individual or contextual differences to induce hormonal responses that are more similar across individuals.

We close this section on oxytocin with a brief discussion of our finding that male participants had higher salivary oxytocin concentrations than female participants (by 1.7 times on average). Importantly, this results does not reflect simple methodological error: our experimental procedures were the same for females and males, and their saliva samples were equally distributed across assays. We also emphasize that although oxytocin has often been associated with female-specific behaviors and effects—e.g., in the context of mothering, parturition, and lactation—it clearly functions in social behavior and cognition (as well as metabolism, cardiovascular function, and bone regeneration) across both females and males, with evidence of overlapping effects, as well as female- and male-specificity (Caldwell, 2018; Quintana and Guastella, 2020). Simple rules about oxytocin and sex differences remain “very difficult ” to state (Caldwell, 2018), and evidence that oxytocin responses and reactions can be more pronounced in males is not unusual (Dumais et al., 2016; Feldman et al., 2010; Herzmann et al., 2013; Lynn et al., 2014; Rilling et al., 2014; Theodoridou et al., 2013). Nevertheless, our finding of higher salivary oxytocin concentrations in males contrasts with at least one other result in the human oxytocin literature. In their study of male and female medical staff at an Italian hospital (*n* = 90, 45 female), Marazziti et al. (2019) found that blood oxytocin concentrations were ~ 2.8 times higher in females compared to males. In considering this sex difference together with our own, it is apparent that neither Marazziti et al. nor the present study examined random samples. This leads to two hypotheses about the sex difference that we observed. One is that male chorists are a “special” group that exhibits higher levels of oxytocin activity (reflected in salivary concentrations). Modern choirs tend to be female-biased, and relative to males who are not in choirs, males in choirs tend to be more accepting of atypical gender behavior and identify less with gender stereotypes like “guys are physical” and “girls are feminine” (Nannen, 2017), potentially reflecting differences in trait empathy. A second hypothesis is that male oxytocin activity is specifically sensitive to variation in opportunities to affiliate with females. Our choir was 63% female (*n* = 71, 45 female) and young (mean age = 23). These circumstances may have led males to feel relatively safe and sociable in support of affiliation and courtship (some may have even have been “in love”). Although most previous endocrine studies of group singing have not included sex as predictor, some insight into evaluating these two hypotheses comes from Schladt et al. (2017). Despite looking, Schladt et al. did not find a significant sex difference in salivary oxytocin in their choir, which was more balanced in terms of participant sex at 55% female (*n* = 38, 21 female). This is a strike against the ‘male chorists are special’ hypothesis, but not necessarily the ‘affiliative opportunity’ hypothesis. If male chorists generally have higher levels of oxytocin activity, this should have been apparent also in Schladt et al. (2017), despite the more balanced choir. In contrast, if male oxytocin activity partially reflects male-female ratios that favor opportunities for males to affiliate with females, the absence of a sex difference in Schladt et al. (2017) would be predicted on the basis of their more balanced choir. Accordingly, we favor increased opportunities for males to affiliate with females as a preliminary explanation of the sex difference that we observed. However, we emphasize that the two hypotheses described above are not mutually exclusive, and that more research on salivary and blood oxytocin concentrations in the general population is needed before the sex differences found here and elsewhere can be interpreted in broader context.

### 4.4. Caveats

The most important caveat here concerns our evolving understanding of the oxytocin system and the aspects of its function that may be assessed in saliva. The principal neural sources of oxytocin—magnocellular and parvocellular neurons in the hypothalamus—exert their effects through at least three different release mechanisms. These include neurosecretory release from magnocellular neurons into peripheral circulation via projections to the neurohypophysis, somato-dendritic release into central extracellular and/or cerebrospinal fluid from magnocellular neurons for paracrine action in the central nervous system, and release from magnocellular and parvocellular projections onto specific central targets in the forebrain, hypothalamus, brainstem, and spinal cord (Jirikowski, 2019; Jurek and Neumann, 2018; Knobloch et al., 2012; Lewis et al., 2020; Ludwig and Leng, 2006; Menon et al., 2018). These mechanisms may act alone or in concert with the others to exert a diversity of influences on social function in different contexts (Jurek and Neumann, 2018; Landgraf and Neumann, 2004). Recent evidence from mice indicates that central synaptic release of oxytocin onto dopaminergic neurons in the reward system is particularly important for understanding affiliation in “consociate” (non-reproductive) relationships (Dölen et al., 2013; Gunaydin et al., 2014; Hung et al., 2017), which may thus provide an appropriate model for relationships between chorists in our study. If comparable central oxytocin projections are similarly key to human consociate affiliation, detection of their activity in saliva seems dubious. Related to this issue, it should be noted that oxytocin's mode of entry into saliva is poorly understood (MacLean et al., 2019; Martin et al., 2018). Like other hydrophilic peptides, oxytocin does not easily cross the blood brain barrier. This can create differences in oxytocin concentrations between the central nervous system and the periphery (McEwen, 2004; Neumann and Landgraf, 2012). Salivary oxytocin is typically interpreted as an indicator of peripheral levels—presumably on the basis of correlations with oxytocin concentrations in blood (Grewen et al., 2010; White-Traut et al., 2009), and because saliva also lies outside the blood brain barrier—but the matter is contested. In particular, two studies conducted in critically ill patients have reached different conclusions, one finding moderate to strong correlations between oxytocin concentrations measured in saliva and cerebrospinal fluid (Martin et al., 2018), and the other indicating very weak correlations (except in the morning; Kagerbauer et al., 2019). The above points about oxytocin and the oxytocin system emphasize that our experimental effects on salivary oxytocin concentrations should be interpreted with caution, as their relation to central oxytocin mechanisms remains unclear. A second caveat concerns the absolute salivary oxytocin concentrations observed in our study (*mean* = 112 pg/ml across all 200 measurements; see Table 1). Although these concentrations are consistent with those reported by previous studies that have used the same Arbor Assays oxytocin ELISA (e.g., Akimoto et al., 2018; Erickson et al., 2020; Leeds et al., 2020), they are considerable higher than most results in the literature. For example, the absolute oxytocin concentrations in saliva measured using another popular oxytocin ELISA (originally produced by Assay Designs Inc., now Enzo Life Sciences Inc.) are more typically in the 5–20 pg/ml range (e.g., Blagrove et al., 2012; Feldman et al., 2010; Grewen et al., 2010; Holt-Lunstad et al., 2011). The reason for the order of magnitude difference between concentrations reported using these two oxytocin ELISAs is unclear. They use different antibodies and protocols. Both now recommend and specify procedures for extracting samples, though it should be noted that these procedures are designed for blood rather than saliva. In a recent review of the challenges faced in measuring oxytocin, MacLean et al. (2019) propose that discrepant results in the literature are due to different methods of sample preparation and measurement being differentially sensitive to “diverse conformational states of the oxytocin molecule” (see also Brandtzaeg et al., 2016; Gnanadesikan et al., 2021). This would indicate that differences in the absolute concentrations of oxytocin reported using different methods reflect real differences in oxytocin biochemistry, rather than the relative validity or invalidity of particular assays. In accord with this perspective, and in defense of our results, we provide the following rationale in support of our oxytocin data having value for examining relative concentration changes across our experimental conditions, despite ongoing debate over absolute values. First, we used the same procedure for all oxytocin measurements; second, the correlation between different measurements of the same participant was *r* = 0.83; and third, the patterns of change that we report are quite similar to those reported by several other recent studies of group singing of similar design (Fancourt et al., 2016; Schladt et al., 2017). A final caveat is that singing and speaking may result in differences in salivary flow rate that could potentially influence measures of salivary oxytocin. Although we are not aware of any evidence that singing and speaking differ in this way, future endocrine studies of group singing that use salivary assays should record the volumes of collected samples so that flow rates may be determined.

## 5. Conclusion

Together with previous studies, our results indicate that group singing in recreational contexts is associated with decreases in the salivary concentrations of oxytocin and cortisol, though we note that the latter effect has not yet been adequately separated from diurnal cycling. More importantly, we additionally found that singing is associated with significantly smaller decreases in salivary oxytocin than speaking—a comparable but non-musical behavior—resulting in concentrations that are significantly higher after singing together compared to speaking together, after controlling for baseline differences. Finally, we found that singing stimulates greater positive shifts in self-reported affect than speaking (particularly when performed together), and that singing together enhances feelings of social connectedness to a greater degree than speaking together. These results provide evidence that singing together can have biological and psychological effects that are associated with an increased capacity for affiliation and social bonding. That said, variation across studies indicates that these effects are secondary to those of broader contextual factors that are likely to shape social dynamics more directly, such as stress levels, the intimacy of interactions, and the status of existing relationships. This implies that deriving further insights into the biology of music from studies focused on its social consequences will require further centering of the naturalistic contexts in which music is proposed to have its most profound effects, e.g., in strengthening the bonds of small interdependent communities, regulating affect, and engaging infants.

## Supplementary Material

Supplementary Material 2.

Supplementary Material 1.

## Figures and Tables

**Table 1:** Overview of hormone data. Means ± standard errors and number of observations (in parentheses) for salivary hormone concentrations, organized by experimental condition.

Hormone	Sex	Singing together		Speaking together		Singing alone		Speaking alone		
						
		before	after	before	after	before	after	before	after	Totals

Oxytocin (pg/ml)	All	106.3±32.8 (32)	73.9±9.7 (33)	189.2±60.3 (30)	85.5±15.2 (28)	96.0±28.8 (20)	61.1±9.1 (23)	190.6±73.3 (18)	112.4±29.7 (16)	122.3±13.4 (200)
	Female	60.3±5.7 (21)	55.2±5.7 (20)	131.1±42.7 (20)	66.5±5.9 (18)	46.7±7.8 (11)	45.9±7.7 (13)	176.3±107.5 (11)	107.0±39.0 (10)	83.1±12.5 (124)
	Male	194.0±91.8 (11)	102.7±21.2 (13)	305.5±158.8 (10)	119.9±40.3 (10)	156.2±58.9 (9)	80.9±16.9 (10)	213.2±93.3 (7)	121.5±44.9 (6)	160.0±28.1 (76)
Cortisol (ng/ml)	All	2.90±0.39 (35)	2.02±0.28 (35)	2.42±0.26 (27)	1.80±0.18 (29)	2.57±0.35 (22)	1.98±0.47 (21)	2.33±0.49 (18)	1.58±0.25 (18)	2.24±0.12 (205)
Testosterone (pg/ml)	Male	78.5±5.4 (10)	77.9±7.5 (11)	85.9±11.0 (9)	73.8±7.9 (8)	74.8±12.8 (9)	76.4±11.6 (10)	68.3±10.6 (6)	61.2±18.3 (5)	75.9±3.5 (68)

**Table 2:** Oxytocin model results. Estimates, standard errors, 95% confidence intervals, post-hoc likelihood ratio tests, and minimum and maximum estimates obtained during model stability assessments for the reduced oxytocin linear mixed model [specified as log_e_(oxytocin) ∼ time * vocal.mode + social.context + sex + test.order + (1 | individual) in R]. Empty cells indicate post-hoc tests that were not performed due to a significant higher-order interaction, or because the effect confounds samples taken before and after experimental manipulations. Asterisks indicate statistical significance at an α-level of 0.05 after Holm-Bonferroni correction for three comparisons. Reference levels for the terms in interactions are the same as those specified in parentheses for main effects. *s.e.* = standard error, *c.i.* = confidence interval, *d.f.* = degrees of freedom.

Effect	Estimate	s.e.	Lower c.i.	Upper c.i.	χ2	d.f.	p	min.	max.

Intercept	4.304	0.156	4.016	4.627				4.212	4.353
Vocal mode (reference = speaking)	−0.394	0.158	−0.696	−0.126				−0.459	−0.299
social context (alone)	0.138	0.063	0.018	0.252				0.113	0.185
Time (before)	−0.433	0.081	−0.599	−0.263				−0.463	−0.384
Test order (first)	0.014	0.063	−0.102	0.135	0.052	1	0.820	−0.016	0.060
Sex (female)	0.515	0.155	0.214	0.815	10.222	1	0.001*	0.435	0.571
Time × vocal mode	0.265	0.111	0.042	0.477	5.566	1	0.018*	0.215	0.296

**Table 3:** Cortisol model results. Results for the reduced cortisol linear mixed model [specified as *log_e_(cortisol) ∼ time + vocal.mode + social.context + sex + test.order + (1 | individual)* in R]. Empty cells indicate post-hoc tests that were not performed because the effect confounds samples taken before and after experimental manipulations. Format is the same as in Table 2. Asterisk indicates significance at an α-level of 0.05 after Holm-Bonferroni correction for three comparisons.

Effect	Estimate	s.e.	Lower c.i.	Upper c.i.	χ2	d.f.	p	min.	max.

Intercept	0.700	0.124	0.469	0.948				0.635	0.739
Vocal mode (reference = speaking)	0.142	0.131	−0.112	0.384				0.102	0.177
Social context (alone)	0.098	0.045	0.06	0.184				0.063	0.123
Time (before)	−0.349	0.038	−0.424	−0.278	65.026	1	<0.001*	−0.357	−0.343
Test order (first)	−0.089	0.045	−0.175	−0.004	3.801	1	0.051	−0.113	−0.056
Sex (female)	0.189	0.137	−0.076	0.457	1.858	1	0.173	0.130	0.242

**Table 4:** PANAS model results. Results for the full PANAS generalized linear mixed model [specified as PANAS.total.affect.transformed ∼ time * vocal.mode * social.context + sex + test.order + (1 | individual) in R]. Empty cells indicate post-hoc tests that were not performed due to a significant higher-order interaction, or because the effect confounds samples taken before and after experimental manipulations. Format is the same as in Tables 2–3. Asterisk indicates significance at an α-level of 0.05 after Holm-Bonferroni correction for three comparisons.

Effect	Estimate	s.e.	Lower c.i.	Upper c.i.	χ2	d.f.	p	min.	max.

Intercept	0.923	0.142	0.632	1.212				0.889	0.980
Vocal mode (reference = speaking)	0.044	0.151	−0.260	0.336				−0.020	0.095
Social context (alone)	0.061	0.123	−0.188	0.305				0.017	0.098
Time (before)	0.073	0.129	−0.201	0.313				0.046	0.098
Test order (first)	−0.094	0.060	−0.216	0.021	2.427	1	0.119	−0.121	−0.072
Sex (female)	−0.050	0.100	−0.238	0.160	0.253	1	0.615	−0.091	−0.008
Vocal mode × social context	−0.108	0.162	−0.432	0.204				−0.147	−0.064
Time × vocal mode	0.176	0.172	−0.148	0.557				0.141	0.263
Time × social context	−0.386	0.162	−0.686	−0.059				−0.428	−0.326
Time × vocal mode × social context	0.729	0.220	0.275	1.145	10.540	1	0.001*	0.657	0.772

**Table 5:** IOS model results. Results for the full IOS cumulative link mixed model [specified as IOS.score ∼ vocal.mode * time + sex + test.order + (1 | individual) in R]. “≤x” = the cumulative log-odds of getting a score of x or lower, i.e., Logit[p(j ≤ x)]. Format is the same as in Tables 2–4. Asterisks indicate significance at an α level of 0.05 after Holm-Bonferroni correction for three comparisons.

Effect	Estimate	s.e.	Lower c.i.	Upper c.i.	χ2	d.f.	p	min.	max.

≤1	−4.688	1.753	−7.679	−1.761				−4.749	−4.432
≤2	−1.692	1.361	−4.627	0.832				−1.755	−1.426
≤3	0.492	1.311	−2.336	3.682				0.255	0.785
≤4	2.602	1.344	−0.244	6.447				2.394	3.178
≤5	5.572	1.469	2.562	10.101				5.362	6.369
≤6	11.172	2.133	8.224	17.574				10.918	12.529
Vocal mode (reference = speaking)	0.159	0.874	−1.731	1.881				0.030	0.337
Time (before)	0.350	0.541	−0.830	1.855				0.160	0.648
Test order (first)	2.203	0.910	0.106	4.911	6.158	1	0.013*	2.101	2.651
Sex (female)	0.238	0.834	−1.615	2.227	0.082	1	0.755	0.168	0.407
Time × vocal mode	1.946	0.751	0.680	3.571	7.058	1	0.008*	1.825	2.222

**Table 6:** Heart rate model results. Results for the full heart rate linear mixed model [specified as heart.rate ∼ vocal.mode * social.context + (1 | individual) in R]. Format is the same as in Tables 2–5. Asterisk indicates significance at an α-level of 0.05.

Effect	Estimate	s.e.	Lower c.i.	Upper c.i.	χ2	d.f.	p	min.	max.

Intercept	76.391	4.927	66.638	85.949				71.639	79.526
Vocal mode (reference = speaking)	16.409	7.175	3.221	30.970				10.630	21.161
Social context (alone)	18.432	3.504	11.507	25.147				16.371	20.146
Vocal mode × social context	−11.832	4.750	−21.368	−2.632	4.793	1	0.0286*	−15.464	−8.700

## Footnotes

2 Here and below, tildas indicate estimates based on a graphical analysis of published figures; conversely, no tilde indicates that published concentration values allowed more precise calculation

3 It should be noted that Grape et al. and Keeler et al. also differ from the other hormonal studies of group singing discussed here because they measured oxytocin in blood rather than saliva. Whether this is the reason for their observation of oxytocin increases as opposed to decreases is uncertain (see “Caveats”).
