# Supplementary material for: Endogenous oxytocin, cortisol, and testosterone in response to group singing
Source: Horm Behav. Author manuscript; Available in PMC 2022 Mar 11. (PMC8915780; doi:10.1016/j.yhbeh.2021.105105)
Supplement: Supplementary Material 1. [file NIHMS1778436-supplement-Supplementary_Material_1_.docx]

SUPPLEMENTARY MATERIAL

for

Bowling et al. Endogenous oxytocin, cortisol, and testosterone in response to group singing

­­

*Contents:*

Supplementary Figure 1. Correlation Analysis

Supplementary Text 1. Baseline Oxytocin Model

Supplementary Data Key. Description of Full Data Set.


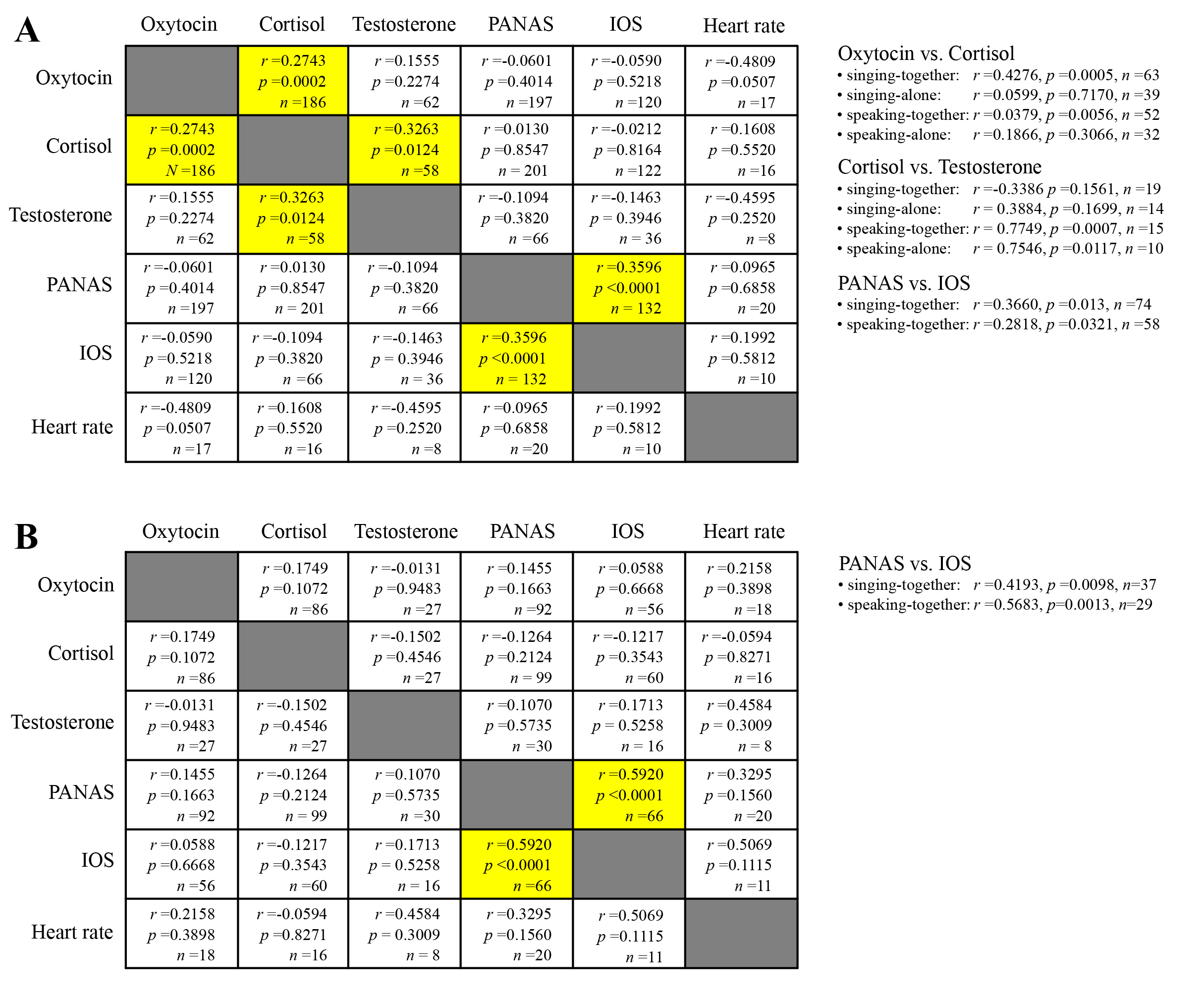


*Supplementary Figure 1. Correlation Analysis.* (A) Matrix of correlations for every pair of dependent variables in this study. Salivary oxytocin and cortisol values were log-transformed prior to testing. For correlations with heart rate (which had only one mean value per condition) values from after the corresponding experimental manipulations for each other variable were used. Yellow cells highlight significant relationships at an *α-*level of 0.05. The text to the right side of the correlation matrix breaks down each significant relationship by experimental condition. (B) A correlation matrix showing the relationships between changes from before to after the experimental manipulations for every pair of dependent variables in this study (for heart rate, mean values were correlated with changes in other variables). Changes were calculated as after minus before. *r* is *Pearson’s* *r*, *p* is the associated *p* value, and *n* is the number of observations. PANAS = Positive And Negative Affect Schedule; IOS = Inclusion of Other in the Self scale. See Supplementary Data 1 for full data set, which can be used to plot any of the above relationships.

*Supplementary Text 1. Baseline Oxytocin Model*

The baseline oxytocin linear mixed model was designed to predict post-experimental salivary oxytocin concentrations as a function of the experimental factors, control predictors, baseline oxytocin concentrations, and a random intercept for individual. The model was specified in R as *log_e_(post.oxytocin) ~ vocal.mode * social.context + pre.oxytocin + sex + test.order + (1 | individual)*, with *post.oxytocin* representing salivary oxytocin concentrations measured after the experimental manipulations, and *pre.oxytocin* representing salivary oxytocin concentrations measured before the experimental manipulation (*pre.oxytocin* values were log-transformed and scaled by subtracting their mean and dividing by their standard deviation, as is standard in co-variate analyses). This baseline modelling approach is less robust than the original approach described in the methods and results of the main text because the number of observations per estimated effect is lower (10.33 here vs. 16.67 for the original model), and the resulting fit is “singular” (indicating that the random effect structure may be overly complex given the data). Nevertheless, model validation procedures indicated that the baseline oxytocin model satisfied assumptions of normally distributed and homogenous residuals, had no issues with collinearity (variance inflation factor =1.31), and had acceptable stability (see table below).

| *effect* | *estimate* | *s.e.* | *lower c.i.* | *upper c.i.* | *χ2* | *d.f.* | *p* | *min.* | *max.* |
| --- | --- | --- | --- | --- | --- | --- | --- | --- | --- |
| intercept | 4.045 | 0.150 | 3.730 | 4.318 |  |  |  | 3.995 | 4.137 |
| vocal mode (reference =speaking) | -0.123 | 0.127 | -0.367 | 0.137 |  |  |  | -0.169 | -0.080 |
| social context (alone) | -0.237 | 0.116 | -0.463 | -0.001 |  |  |  | -0.308 | -0.208 |
| baseline oxytocin | 0.517 | 0.043 | 0.438 | 0.604 | 141.334 | 1 | <0.001* | 0.501 | 0.534 |
| sex (female) | 0.032 | 0.085 | -0.126 | 0.184 | 0.137 | 1 | 0.711 | 0.005 | 0.067 |
| test order (first) | 0.128 | 0.080 | -0.026 | 0.287 | 2.548 | 1 | 0.113 | 0.091 | 0.152 |
| vocal mode × social context | 0.384 | 0.157 | 0.078 | 0.706 | 6.000 | 1 | 0.016* | 0.348 | 0.444 |

*Baseline oxytocin model results.* Estimates, standard errors, 95% confidence intervals, post-hoc likelihood ratio tests, and minimum and maximum estimates obtained during model stability assessments for the baseline oxytocin linear mixed model. Empty cells indicate post-hoc tests that were not performed due to a significant higher-order interaction. Asterisks indicate statistical significance at an *α*-level of 0.05 after Holm-Bonferroni correction for four comparisons. Reference levels for the terms in interactions are the same as those specified in parentheses for main effects. *s.e.* = standard error, *c.i.* = confidence interval, *d.f.* = degrees of freedom.

The baseline oxytocin model was a significantly better predictor of post-experiment salivary oxytocin concentrations compared to a parallel null model that was the same except that it lacked effects for vocal mode, social context, and their interaction (*χ^2^* =7.85, *d.f.* =3, *p* =0.049). As might be expected, post-hoc tests indicated a significant main effect of baseline oxytocin concentrations (*estimate* ±*s.e.* =0.517 ±0.043; *χ^2^* =78.7, *d.f.* =1, *p* <0.001). Post-hoc tests additionally showed that the interaction between vocal mode and social context was significant (*estimate* ±*s.e.* =0.384 ±0.157; *χ^2^* =6.0, *d.f.* =1, *p* =0.016). This interaction is shown in the figure below, which plots the results of the baseline oxytocin model as a function of vocal mode and social context, drawn with the effects of baseline oxytocin, test order, and sex centered.


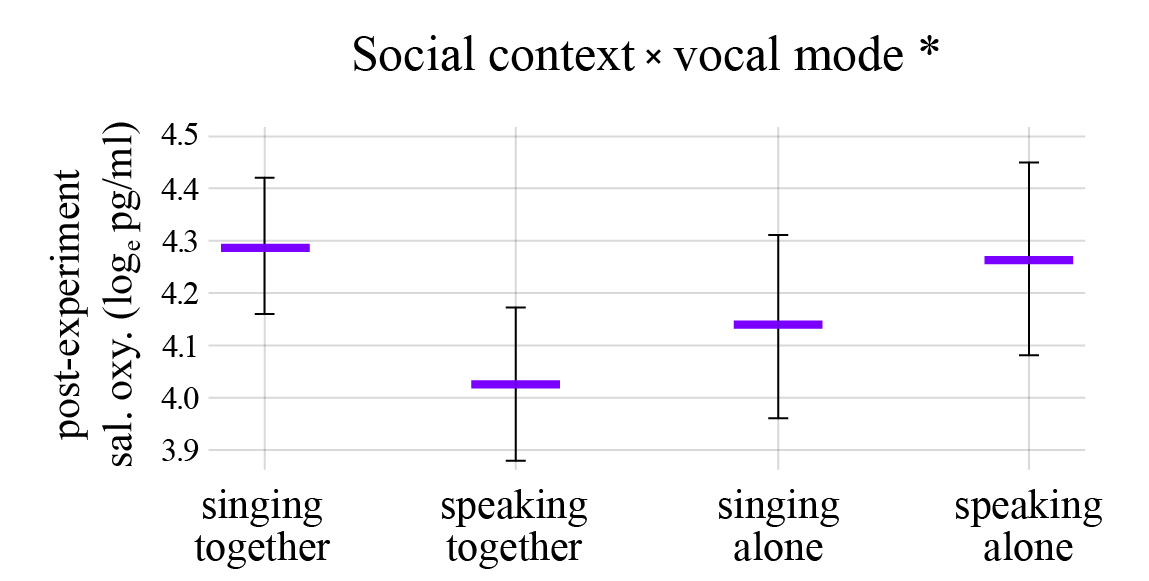


According to this plot, we further examined three contrasts to determine significant pairwise differences. These contrasts were: (1) singing together versus speaking together (*mean* =72.7 pg/mL [*c.i.* =64.1-83.1] vs. 56.0 [48.4-64.9], *t*[100.5] =-2.497, *p* =0.014); (2) speaking together versus speaking alone (56.0 [48.4-64.9] vs. 71.0 [59.2-85.6], *t*[65.7] =1.941, *p* =0.057); and (3) speaking alone versus singing alone (71.0 [59.2-85.6] vs. 62.8 [52.5-74.5], *t*[100.5] =0.919, *p* =0.360). Only the difference between singing together and speaking together is significant. This is true whether or not the associated p-value is corrected for multiple comparisons.

It is worth noting that, in contrast with the original oxytocin model, the effect of sex in the baseline oxytocin model was not significant (see table above). This suggests that the sex difference in salivary oxytocin concentrations reported in the main text was primarily due to differences between males and females at baseline (this can be confirmed by inspecting Figure 2A). Sex did not interact with the two-way interaction between vocal mode and social context in the baseline oxytocin model (i.e., testing the three-way interaction between sex, time, and vocal mode showed that it was not significant, *χ^2^* =0.011, *d.f.* =1, *p* =0.918). This indicates that the interaction between vocal mode and social context observed in the baseline oxytocin model was similar for males and females.

Finally, with respect to the potential cause(s) of baseline differences in salivary oxytocin between conditions, we note that our experimental protocol did not explicitly prevent participants from inferring their condition assignment prior to the time at which baseline saliva samples were taken. Accordingly, participants assigned to speak (the more unusual condition) may have mounted an anticipatory oxytocin response, perhaps in preparation for focused interactions within a new subgroup (when speaking-together), or as part of a reaction to being selected for a novel activity (when speaking-alone). Regardless of whether or not anticipatory responses were the true cause of the baseline differences in salivary oxytocin that we observed—it is also possible that they were a product of random chance—future studies can do better by prioritizing stricter limits on information available to participants.

All procedures related to the baseline oxytocin model were performed in R using the same software and packages described in the main text. Additionally, the pairwise contrasts made here were performed using the “lsmeans” function from the R package “lsmeans” (Lenth, 2016).

References:

Lenth R.V. (2016). Least-Squares Means: The R Package lsmeans. Journal of Statistical Software, 69(1), 1-33.

*Supplementary Data 1. Full Data Set.*

All of the data used in this study is provided in the “choir_data.xlsx” file.

In the “Hormones and Surveys” sheet, each row represents one saliva sample or survey. The columns correspond to the following variables:

- “subject.ID” = a unique numerical identifier for each participant.
- “time” = whether a saliva sample or survey was collected “before” or “after” the corresponding experimental manipulations
- “vocal.mode” = whether a saliva sample or survey was obtained from a “singing” condition or a “speaking” condition
- “social.context” = whether a saliva sample or survey was obtained from an “alone” condition or “together” condition
- “sex” = whether the participant identified as male or female.
- “oxytocin (pg/ml)” = the measured concentration of salivary oxytocin.
- “cortisol (ng/ml)” = the measured concentration of salivary cortisol.
- “testosterone (pg/ml)” = the measured concentration of salivary testosterone.
- “PANAS” = Untransformed total affect scores from the Positive And Negative Affect Schedule
- “IOS” = Scores from the Inclusion of Other in the Self scale.

In all columns “NA” indicates missing data (due to corrupted samples, incomplete surveys, or because the measure was not taken in the indicated condition).

In the “Heart Rate” sheet, each row represents one average heart rate data point. The columns are the same as above with the exception of:

- “mean (bpm)” = mean heart rate in beats per minute, calculated over the middle 10 minutes of the indicated experimental condition.
- “s.d.” = standard deviation of heat rate in beats per minute, calculated over the middle 10 minutes of the indicated experimental condition.
